# An overview of *STRUCTURE*: applications, parameter settings, and supporting software

**DOI:** 10.3389/fgene.2013.00098

**Published:** 2013-05-29

**Authors:** Liliana Porras-Hurtado, Yarimar Ruiz, Carla Santos, Christopher Phillips, Ángel Carracedo, Maria V. Lareu

**Affiliations:** ^1^Universidad Tecnológica de PereiraPereira, Colombia; ^2^Forensic Genetics Unit, Institute of Legal Medicine, University of Santiago de CompostelaSantiago de Compostela, Spain; ^3^Genomics Medicine Group, CIBERER, University of Santiago de CompostelaSantiago de Compostela, Spain

**Keywords:** *STRUCTURE*, *CLUMPP*, *distruct*, *STRAT*, population structure, case-control association studies, stratification

## Abstract

**Objectives:** We present an up-to-date review of *STRUCTURE* software: one of the most widely used population analysis tools that allows researchers to assess patterns of genetic structure in a set of samples. *STRUCTURE* can identify subsets of the whole sample by detecting allele frequency differences within the data and can assign individuals to those sub-populations based on analysis of likelihoods. The review covers *STRUCTURE's* most commonly used ancestry and frequency models, plus an overview of the main applications of the software in human genetics including case-control association studies (CCAS), population genetics, and forensic analysis. The review is accompanied by supplementary material providing a step-by-step guide to running *STRUCTURE*.

**Methods:** With reference to a worked example, we explore the effects of changing the principal analysis parameters on *STRUCTURE* results when analyzing a uniform set of human genetic data. Use of the supporting software: *CLUMPP* and *distruct* is detailed and we provide an overview and worked example of *STRAT* software, applicable to CCAS.

**Conclusion:** The guide offers a simplified view of how *STRUCTURE*, *CLUMPP*, *distruct*, and *STRAT* can be applied to provide researchers with an informed choice of parameter settings and supporting software when analyzing their own genetic data.

## An overview of the *STRUCTURE* program

*STRUCTURE* is a freely available program for population analysis developed by Pritchard et al. (2000a). *STRUCTURE* analyses differences in the distribution of genetic variants amongst populations with a Bayesian iterative algorithm by placing samples into groups whose members share similar patterns of variation. *STRUCTURE* both identifies populations from the data and assigns individuals to that population representing the best fit for the variation patterns found. Typically *STRUCTURE* is the first step in examining population structures that emerge from the sample set to provide a preamble to further genetic analysis or to infer the origins of individuals with unknown population characteristics, especially when population admixture has occurred. As *STRUCTURE* uses the core Bayesian principle of comparing likelihoods, prior information about study samples can be supplied to further shape the analysis. For example, information about sampling location can be input—a characteristic, if shared between individuals that can be associated with their genetic proximity. The definition of populations can be assessed from geographical distribution, but is also often based on alternative criteria, including the phenotype, behavior, and ecology of sampled individuals, while linguistic and cultural characteristics can also define human populations. Therefore, it is important to assess whether assignment of individuals to populations with non-genetic criteria is consistent with genetic patterns detected between populations (Pritchard et al., 2000a; Jobling et al., 2004; Waples and Gaggiotti, 2006).

*STRUCTURE* uses a systematic Bayesian clustering approach applying *Markov Chain Monte Carlo* (MCMC) estimation. The MCMC process begins by randomly assigning individuals to a pre-determined number of groups, then variant frequencies are estimated in each group and individuals re-assigned based on those frequency estimates. This is repeated many times, typically comprising 100,000 iterations, in the burnin process that results in a progressive convergence toward reliable allele frequency estimates in each population and membership probabilities of individuals to a population.

Measurement of the assumed number of populations uses the MCMC estimation and is performed separately from the burnin. *STRUCTURE* performs individual analyses for each assumed population number from one to a reasonably appropriate number for the sampling regime. *STRUCTURE* applies a model to the data of *K* assumed populations or genetic groups, each characterized by a subset of allele frequencies identified in the data. Commonly *K* is not readily defined by the user for the sample set, although this parameter must be pre-selected. Therefore, an appropriate first step is to calculate the likelihood of the data for a range of *K* values by creating posterior probabilities of *K*, termed *X* and written: *X*|*K*. Since *K* is not an absolute value, user-defined values should be considered carefully, taking into account characteristics of the sampled populations. Running a range of prescribed *K* settings to obtain their *X* values normally creates probabilities smaller than those for the most appropriate *K* value but beyond this probabilities tend to be very similar for higher *K* values. Therefore, plots of *X* values typically progress to a plateau for levels of *K* beyond the most applicable number of detected populations, so the smallest stable *K* value represents the optimum value. Kalinowski (2011) notes that better clusters are created applying the most realistic *K* values, so it is prudent to obtain the smallest value of *K* that maximizes the global likelihood of data—an approach capturing the major underlying population structure in the data without overestimating it.

During each analysis membership coefficients summing to one are assigned to individuals for each group. The membership coefficient matrix, termed the *individual Q-matrix*, is generated with rows for the number of individuals analysed and columns for *K* clusters. The average individual membership coefficients to each population form the *population Q-matrix*. If admixture is not a factor for the population samples analysed, posterior probabilities of belonging to each of *K* groups are calculated for each individual and a sample can be considered a member of the group with the highest probability. If admixture is considered membership coefficients are made across multiple clusters.

Bayesian population analysis methods equate allele frequencies that define the population and the frequencies found in individuals identified as originating from that population. Therefore, the ability of Bayesian methods to differentiate populations amongst a set of samples is severely restricted from limited sample sizes and small marker numbers (Corander et al., 2003; Corander and Marttinen, 2006). Genetic markers applied to *STRUCTURE* analyses ideally show selective neutrality, low mutation rates and absence of linkage disequilibrium (LD) (Pritchard et al., 2000a; Corander et al., 2003). In short, they are treated as independent variables and so-called naïve Bayesian approaches assume independence, but without a guarantee this applies. However, enhancements since *STRUCTURE* 2.3.1 permit inclusion of weakly linked markers with some degree of non-independence (Falush et al., 2003). Additionally, Falush et al. (2007) describe an algorithm that allows application of all available models to dominant markers (loci often characterized by genotype ambiguity).

Note that SNPs (single nucleotide polymorphisms), as binary markers, have lower variability than multiple-allele loci, requiring much smaller sample sizes to obtain accurate allele frequency estimates. Shi et al. (2010) suggested population samples as low as four individuals are sufficient to provide reliable data by demonstrating subsamples of four taken from much larger population samples give very similar posterior median population parameters. Microsatellites require considerably larger samples sizes than SNPs to reliably capture patterns of variability in a population. Lastly, recent forensic guidelines for microsatellite population surveys recommend genotyping a minimum 500 samples (Carracedo et al., 2013).

## Ancestry and allele frequency models

Two terms are relevant to a review of the estimation of ancestry: local ancestry and global ancestry. Local ancestry estimates the extent to which each person's genome is divided into chromosome segments of definite ancestral origin. Global ancestry estimates the proportion of ancestry from each contributing population, considered as an average over the individual's entire genome (Alexander et al., 2009). *STRUCTURE* only estimates global ancestry by implementing different models of population structure to the data. Selection of the most appropriate model depends on the user's data and study objectives. Two ancestry models applied by *STRUCTURE* are the *no admixture* and *admixture* models. If there is no prior knowledge about the origin of the populations under study or if there is reason to consider each population as completely discrete, the *no admixture* model is appropriate. However, admixture between populations is a common characteristic such that a large proportion of sampled individuals can have recent ancestors from multiple populations. In these cases knowing the approximate median value of the ancestral population proportions for each individual and/or their populations of origin is a very useful part of the characterization of the populations under study. In these cases the *admixture* model is more appropriate. Both models can be used with consideration for sampling location information by applying the prior model parameter: *LOCPRIOR* to the population model (Hubisz et al., 2009). This option can be used when there is additional sample-characteristic data available to the user, including: linguistic, geographical, cultural, or phenotypic information. The *LOCPRIOR* parameter is particularly informative when there are weak population structure signals—a situation that can result from using reduced numbers of markers, small sample sizes or due to close relationships between populations.

The third model parameter is *linkage*, based on the *admixture* model and this is designed to deal with admixture LD: the characteristic of extended LD found in admixed populations and often deliberately sought in association studies. This model was outlined by Falush et al. (2003) and provides more accurate estimates of statistical uncertainty when linked markers are used.

*No admixture*, *admixture*, and *linkage* models can also be analysed as part of the *USEPOPINFO* model. This model uses the population labels to calculate the probability that each individual has of originating from the assumed population—individuals with low probabilities can be considered as hybrids or migrants (Pritchard et al., 2000a). This parameter should be used cautiously—applied only when the population labels are well defined beforehand and correspond almost exactly to the groups ultimately defined by the *STRUCTURE* results. The disadvantage of the *USEPOPINFO* model arises with the posterior handling of the results. The *individual Q-matrix* comprises probabilities (and not ancestry membership proportions) that are presented in a format that is incompatible with *post-hoc* data-processing software such as *CLUMPP* or *distruct*.

All the models considered until now can be used in conjunction with an alternative approach to *USEPOPINFO*—the *POPFLAG* model. *POPFLAG* considers the specified information about the population of origin of a portion of the individuals to help infer the ancestry of other samples with unknown origin. This option should also be used with caution because selected samples will be treated as the “reference” set (pre-assigned *POPFLAG* = 1) so allele frequency estimates are based on a reduced sub-set of samples and will directly affect the grouping of the unknown individuals (pre-assigned *POPFLAG* = 0). *POPFLAG* is an artificial model that assesses the individual probability of being part of a particular population, but it can be useful if the objective is to efficiently group individuals/populations by comparison with a particularly well-defined and studied reference data set (Pritchard et al., 2000a). One such reference set, widely applied to human population genetics studies, is the CEPH human genome diversity panel (HGDP-CEPH) (Cann et al., 2002) with the advantage that population structure has been identified in this sample set in a wide range of studies using different markers and a variety of data depths, but with consistent findings (Rosenberg et al., 2002; Enoch et al., 2006; Abdulla et al., 2009). When the *POPFLAG* model is used in conjunction with the *USEPOPINFO* model the *individual Q-matrix* is composed of two distinct parts: for *POPFLAG* = 1 individual's the matrix presents probabilities, while for *POPFLAG* = 0 individuals ancestry membership proportions are given according to the admixture model defined (*no admixture*, *admixture*, or *linkage*).

Related subjects should be detected then excluded from reference data since shared variation is inflated in frequency causing estimation bias that consequently affects analysis of the ancestry of study samples. Prudent checks of reference data are clearly advisable, for example, an audit of the HGDP-CEPH samples was published by Rosenberg (2006) where two atypical individuals, 13 duplicates, and as many as 77 first-degree relative pairs were identified and removed. Related subjects can regularly form part of the study set and this approach is central to linkage studies in genetic epidemiology as these seek loci with correlations between traits of interest and patterns of transmission of DNA sequence over generations in a known pedigree (Astle and Balding, 2009).

Two allele frequency models are available. The *correlated allele frequencies* model assumes a level of non-independence, so is more conservative. The *independent allele frequencies* model requires knowledge about the correlation levels across populations—allele frequencies should be reasonably different in distinct populations. The *correlated allele frequencies* model provides greater power to detect distinct populations that are particularly closely related, although this model will give the same results as the *independent allele frequencies* model in the absence of high levels of correlation across populations. Therefore, it is prudent to use the *correlated allele frequencies* model since this will guarantee that a previously undetected correlation is identified, but without affecting the results if no such correlation exists.

**Figure 1:**
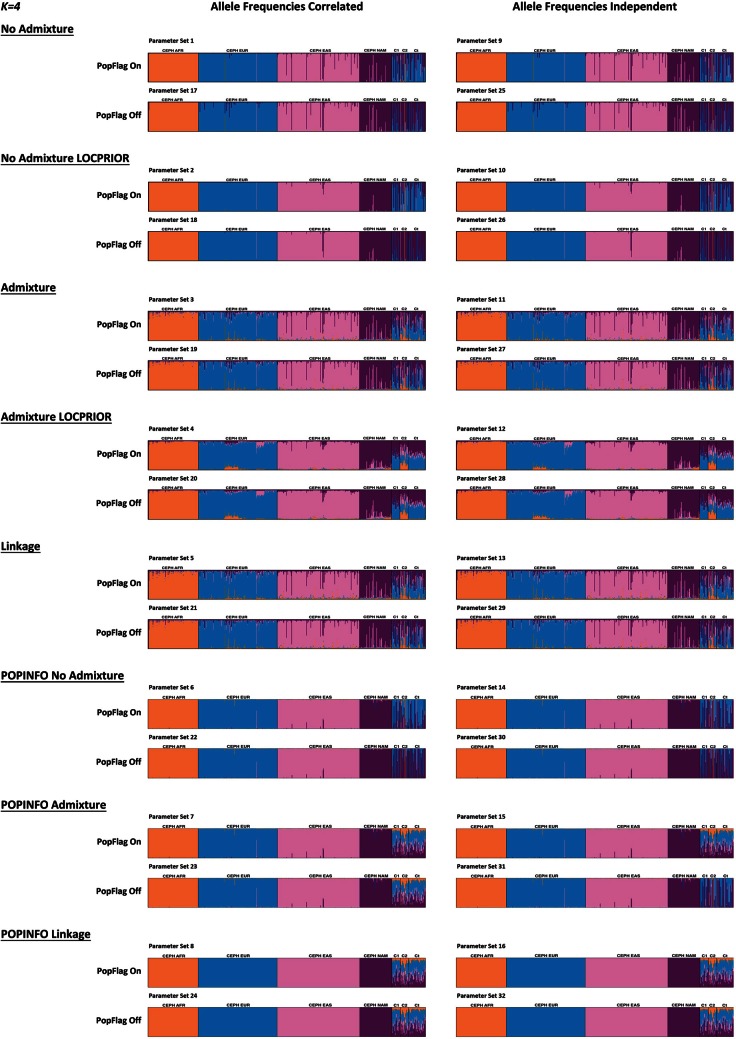
***STRUCTURE* bar plots representing *K* = 4 for the principal analysis parameter combinations available to the user.** These graphics were obtained with *distruct* and using *CLUMPP* to align the three replicates for *K* = 4 (all runs were performed with 100,000 burnin period and 100,000 MCMC repeats after burnin). The exception was the POPINFO parameter sets for which direct *STRUCTURE* bar plot outputs were used. Human genetic data comprised genotypes listed in Table S1 consisting of 100 Africans: CEPH AFR, 158 Europeans: CEPH EUR, 165 East Asians: CEPH EAS, and 64 Native Americans: CEPH NAM from the HGDP-CEPH human diversity panel. An artificial case-control group was created using HapMap Mexican and Puerto Rican samples giving a total 67 sample divided into Cases 1 (C1), Cases 2 (C2), and Controls (Ct). Markers were: 9 AIM-SNPs (two triallelic), 3 phenotype associated SNPs and 5 AIM-SNPs on the X-chromosome. The phenotype and the X-SNPs are linked forming two distinct linkage disequilibrium groups—their genetic distance was used to define linkage disequilibrium groups. Each parameter setting and the results obtained are described in detail in Supplementary Material 1.

Summarizing the range of models that can be applied to a uniform set of genetic data, Figure 1 shows bar plots produced by *STRUCTURE* when applying each of the models described above. The application of each model is outlined in detail in the Supplementary Material 1, section 5.

It is noteworthy that a level of finesse exists when implementing analysis models in *STRUCTURE*. All the models described above include specific statistical parameters that can be adjusted to more sensitive values, including *r* (informativeness of the sampling location data), *alpha* (relative admixture levels between populations), and *lambda* (quantifies the independence between markers in terms of their allelic frequency distribution).

## Assignment of individuals to a population and choice of markers

Assigning individuals to populations is often useful in population genetics studies (Pritchard et al., 2000a) where making a population classification can provide an inference of individual ancestry that may not have been adequately defined beforehand (Royal et al., 2010). The typical approach has already been described for *STRUCTURE*: establishing pre-defined populations from reference samples and assigning individuals of unknown origin to these populations. Reference samples provide allele frequency estimates in each population that are then used to compute the likelihood of membership of samples of unknown origin to any population (Pritchard et al., 2000a).

When using small numbers of markers, highly differentiated genetic variants are more informative per locus than randomly chosen markers. In these cases a measure of marker differentiation or divergence becomes an important factor in selecting markers to type. The informativeness metric *In*, proposed by Rosenberg et al. (2003) is a useful measure of individual divergence per locus and per population comparison that can help guide marker choice ahead of commitments to the necessary genotyping.

The best markers for the inference of ancestry membership proportions are those that efficiently distinguish different populations, i.e., markers showing different alleles at very high frequency in distinct parental populations. Since fixed variation, private to one population, is very rare (Pfaff et al., 2004; Lao et al., 2006) marker selection must always be broadened to loci with maximized allele frequency differences between ancestral populations—these are usually termed Ancestry Informative Markers or AIMs (Yang et al., 2005; Enoch et al., 2006; Salas et al., 2006). Autosomal SNPs are increasingly favored for human population analysis because, in addition to their widespread genomic distribution and ease of genotyping in very dense marker arrays, they are independent of admixture sex bias that routinely affects the distribution of variation of the Y chromosome or mitochondrial DNA. Segregating autosomal markers allow a more thorough measure of admixture in an individual contributed by all of their ancestors rather than just those of single uni-parental lineages (Lao et al., 2006; Phillips et al., 2007; Halder et al., 2008; Royal et al., 2010).

## Reference samples and variation databases

Amongst the objectives of human population genetics is the measurement of population-related parameters (e.g., effective size, degrees of relatedness, effects of local natural selection), the detection of admixture and the reconstruction of past demographic events. Therefore, the proper definition of population structure is a key step in studying the populations of a region. In the case of admixed populations it is particularly important to define the original contributing populations by characterizing reference populations and databases of human variation forming the primary data sources for such studies.

A good starting point for collating human SNP variation data from the most extensive catalogs and for standard reference populations is *SPSmart* [http://spsmart.cesga.es, (Amigo et al., 2008)]. *SPSmart* has the advantage of being inclusive of all current SNP databases, specifically: 1000 Genomes, HapMap, Perlegen, and Universities of the Stanford and Michigan CEPH-HGDP repositories. Additionally *SPSmart* allows the collection of genotype data from a large number of markers at a time and their direct transfer into population analysis programs of choice, including *STRUCTURE* (although some data re-arrangement is necessary to create the input file).

The HGDP-CEPH is frequently used as a panel of population reference samples and CEPH panel samples from the same predefined population analysed with *STRUCTURE* nearly always share similar membership coefficients in inferred clusters (Pritchard et al., 2000a; Rosenberg et al., 2002; Jobling et al., 2004; Enoch et al., 2006; Abdulla et al., 2009). Royal et al. (2010) noted that there are some limitations to the accuracy of ancestry inference within and among regions that may be the result of the incomplete sampling by the CEPH-HGDP of total human genetic diversity (Ashg, 2008). The first study using *STRUCTURE* was performed in 2002 by Rosenberg et al. using 377 microsatellites to infer human population structure in the HGDP-CEPH worldwide population sample. This study concluded that global populations could be grouped into six major discrete ancestral groups matching well with continental distributions. Subsequent studies confirmed that when individuals are grouped on the basis of genetic similarity, group membership corresponds closely to predefined regional or population groups or to collections of geographically and linguistically similar populations (Allocco et al., 2007; Li et al., 2008). In particular, the study of Li et al. (2008) using *FRAPPE*, a very similar alternative population clustering method to *STRUCTURE*, divided the HGDP-CEPH into seven major population groups. Furthermore, such studies indicated it was also possible to infer the ancestry of individuals from recently admixed populations in the context of the contributions of putative parental populations (Yang et al., 2005). Mixed ancestries inferred from genetic data can often be interpreted as arising from recent admixture among multiple founder populations. However, it can also be the result of a shared ancestry before the divergence of the two populations with a lack of subsequent gene flow between them (Li et al., 2008).

## Case-control association studies

Case-control association studies (CCAS) provide powerful strategies to identify loci contributing to complex disease. The simplest approach genotypes markers in samples of cases and unrelated controls then tests these for allele frequency differences at each marker—association of genomic regions indicates the loci they contain are possibly linked to disease susceptibility or the presence or absence of particular phenotypes (Pritchard and Donnelly, 2001). However, presence of population structure between case and control groups can produce confounding effects where high false positive rates from allele frequency differences between subpopulations mimics associations with the studied disease. Cryptic relatedness from undetected kinship amongst study subjects is a further confounding effect with potential to exaggerate false positive rates (Voight and Pritchard, 2005; Astle and Balding, 2009). Its effects can be negligible for well-designed studies in outbred populations but cryptic relatedness is more significant in effect for small and isolated populations, when extensive inbreeding occurs or with bias in sample collection toward relatives (Voight and Pritchard, 2005; Astle and Balding, 2009).

Two main approaches are favored to overcome effects of hidden or cryptic structure when it exists between case and control groups: genomic control (GC) and structured association (SA). Pritchard and Donnelly (2001) reviewed GC methods using chi-square tests to detect population stratification through estimation of increase in the test statistic null distribution compared to those of unlinked markers typed in the same group. Using the adjusted distribution gives corrected *p*-values at any given locus. SA approaches use additional genotype information from unlinked markers to estimate the number of subpopulations and each individual's assignment to these subpopulations. This information can then be used to construct a test for association (Pritchard and Donnelly, 2001). SA methods perform well but are computationally demanding and very reliant on estimating the correct number of subpopulations. The recent development of faster model-based methods such as that implemented by ADMIXTURE makes the application of SA methods more feasible (Price et al., 2010). In comparison, GC methods while faster and more straightforward can lack power in certain scenarios, for example, when the markers used are not informative for population comparisons or in the case of cryptic relatedness (Price et al., 2010).

**Figure 2:**
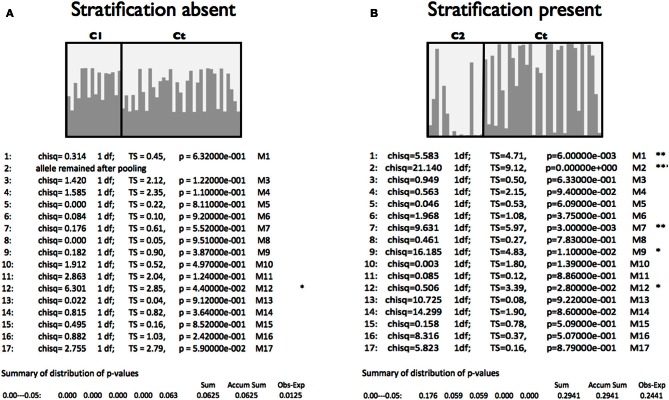
**Example case-control sample analyses comparing scenarios with the presence or absence of stratification**. *STRUCTURE* bar plots and *STRAT* table results are shown. **(A)** Case 1 (C1) are compared to the Control (Ct) samples. **(B)** Case 2 (C2) are compared to the Control (Ct) samples. Details of these analyses are described in Supplementary Material 1.

Procedures based on logistic regression that are flexible, computationally fast, and easy to implement, provide protection against the effects of cryptic substructure, even though not explicitly modeling the population structure (Setakis et al., 2006). However, if there is enough information for reliable estimation of sub-population data, the power and flexibility of SA approaches, facilitated by dedicated software such as *STRAT*, makes them preferable to GC methods (Pritchard and Donnelly, 2001; Price et al., 2008). In fact, analyses performed by Karkkainen and Sillanpaa (2012) demonstrated that in most cases Bayesian multilocus association approaches improve the accuracy of association studies and avoid false positive results. *STRAT* is applicable to association mapping, enabling valid case-control studies even in the presence of population structure. This method was first described by Pritchard et al. (2000b). The application of *STRAT* improves association studies as it takes into account the confounding effects of population stratification using selected panels of AIMs. Figure 2 summarizes typical *STRAT* analyses with stratification between case and control samples vs. stratification absent. *STRAT* chi-square *p*-values are listed for 17 markers directly comparable to *STRUCTURE* bar plots above. This worked example is discussed in detail in Supplementary Material 1, section 5. It should be noted that as well as common SNPs showing stratification, loci with low frequency (1–5%) or rare (≤1%) minor alleles can inflate the false positive rate significantly (Babron et al., 2012; Mathieson and McVean, 2012) while showing different patterns of stratification from the same population comparisons. The implication of the Babron et al. study is that population analyses may be required for each class of SNP variation when high density SNP data is used for the CCAS.

Several studies have applied *STRAT*-based stratification control, when there is doubt about the validity of the associations found (i.e., not spurious associations from population stratification) or when it is known that two or more populations are admixed (Han et al., 2008; Tian et al., 2008). The accuracy of inferences improves with sample size, number of loci, and degree of divergence between populations (Pritchard et al., 2000a). An instructive study by Campbell et al. (2005) analyzed the efficacy of stratification control by constructing a case-control group based on adult height then measuring stratification with 111 random SNPs and 67 AIMs. Both SNP sets failed to detect stratification between case and controls but one SNP in LCT (rs4988235: LCT-13910C→T) with a frequency difference between north and south Europe co-incidental with average height difference across this geographic distance was strongly associated. Re-matching case and control subjects into equivalent numbers of north and south Europeans in each loses the spurious association. Price et al. (2008) developed an AIM panel capable of distinguishing north-west and south-east European ancestry that applied to Campbell's study efficiently detected stratification. These studies demonstrate the importance of careful marker selection, particularly analysing closely related populations.

Finally a further class of model to detect and correct for stratification is the mixed model—this approach should perform better when confounding effects are present since it simultaneously addresses population's stratification, family structure, and cryptic relatedness (Price et al., 2010). Mixed model methods are computationally challenging but the optimization implemented has enabled large datasets typical of GWAS studies to be readily analysed (Zhang et al., 2010).

## Other applications: forensic analysis

Forensic DNA analysis is a powerful tool in near-universal use as a core part of police investigations. DNA profiling is particularly informative used in conjunction with DNA databases of offender profiles. Although situations occur with no database match, so any additional information that can be obtained from the DNA becomes valuable (Lowe et al., 2001). Information indicating probable ancestry of an unknown offender can help direct investigations towards a smaller suspect group (Lowe et al., 2001; Phillips et al., 2011). Likewise interest is growing in the prediction of externally visible characteristics such as eye color (Ruiz et al., 2013; Walsh et al., 2013). Currently microsatellite short tandem repeat (STR) typing is the standard approach and still the method of choice, providing extremely high discrimination power for most problems of human identification (Chakraborty et al., 1999; Butler, 2005). Several studies have concluded STR profiles can enable ancestry inference (Bowcock et al., 1994; Graydon et al., 2009; Londin et al., 2010; Phillips et al., 2011), but only with sufficient reliability when other AIMs such as SNPs are included (Phillips et al., 2011). One example of the benefits from adding specialized marker sets to enhance ancestry analysis in forensic casework is the 11-M Madrid bomb attack investigations where results of a 34-SNP ancestry test were analysed with *STRUCTURE* to infer the probable ancestry of suspects (Phillips et al., 2009). One disadvantage of *STRUCTURE* highlighted by this study is the difficulty of analyzing single genotype profiles. An alternative online classifier termed *Snipper* (Phillips et al., 2007), analyzes single profiles with a near identical Bayesian algorithm and gives likelihoods of membership to ancestry groups inferred from user-defined training sets acting as the reference material.

## Alternative population analysis approaches

Several population analysis programs provide alternatives to *STRUCTURE* and are applicable to most of the analyses outlined above. Comparisons of many of these programs are presented in Table 1. A more comprehensive review by Liu et al. (2013) assesses regularly chosen alternative software and methods for population analysis. Such programs are usually designed for a specific population analysis application so they lack the major advantage of *STRUCTURE* in offering the flexibility to adapt to varying analysis demands. Although many options are available for analysis of population data and inference of genetic ancestry there is no one program applicable to all situations or data types. However, *STRUCTURE* can readily handle different markers and their characteristics (SNPs, STRs, linked markers, and loci with dominance). Provision of different ancestry and allele frequency models allows the user to adapt different analyses in a straightforward way with a unified approach based on *STRUCTURE* alone or combined with supporting programs. The adaptability of *STRUCTURE* is underlined by its widespread application to population genetics studies in general or specifically to forensic analysis and stratification adjustment of CCAS.

**Table 1:** **Alternative population analysis programs and their comparison with *STRUCTURE* software**.

**Software**	**Description**	**Advantages compared to *STRUCTURE***	**Disadvantages compared to *STRUCTURE***	**References**
ADMIXTURE	A program for maximum likelihood estimation of individual ancestries from multilocus SNP genotypes. It allows automated choice of the population number and use of known ancestral populations in a supervised learning mode.	ADMIXTURE uses the same statistical model as *STRUCTURE* but calculates population parameters much more rapidly using a fast numerical optimization algorithm. This allows much larger marker sets to be used (potentially valuable incorrecting for population stratification in CCAS).	Models implemented in ADMIXTURE do not explicitly account for linkage disequilibrium (LD) between markers.	Alexander et al., 2009; Alexander and Lange, 2011; Zhou et al., 2011
ADMIXMAP	A program for modeling admixture, using marker genotypes and trait data on a sample of individuals from an admixed population, where the markers have been chosen to have strongly differentiated allele frequencies between two or more of the ancestral populations contributing to the admixture.	ADMIXMAP models the dependence of the outcome variable on individual admixture and thus it can adjust for the effect of individual admixture on the outcome variable.	Some unnecessary computational components for inference of individual ancestry are included so computation times are longer.	McKeigue et al., 2000; Tang et al., 2005; McKeigue, 2007
		It allows the user to supply prior distributions for the allele frequencies. It allows for allelic association (other than that generated by admixture), and is therefore suitable for analysis of datasets in which two or more tightly linked loci (for instance SNPs in the same gene) have been genotyped.	It does not assume a possible correlation between the allele frequencies in each subpopulation.	
FRAPPE	A maximum likelihood (ML) approach that estimates individual admixture fractions, using SNP or microsatellite genotype data, that allows for uncertainty in ancestral allele frequencies.	The efficiency of FRAPPE is similar to that of MCMC Bayesian approaches but the computation time is much reduced.	The parameter estimates can be slightly inaccurate due to the relaxed convergence criterion that permits fast termination of the algorithm.	Tang et al., 2005; Alexander et al., 2009
		When the ancestral groups are small and the markers are not highly informative it can produce less biased estimates.	FRAPPE does not allow the incorporation of known map information and does not model the LD.	
			It does not provide measures to choose an optimal K value.	
EIGENSOFT	A program suite that has two main components, *EIGENSTRAT* uses principal component analysis to correct for population stratification in association studies and *SMARTPCA* for the detection and analysis of population structure.	In the case of non-admixed populations it has a better performance regarding the inference of population stratification.	The degree of admixture from ancestry fractions is not included in the results.	Patterson et al., 2006; Price et al., 2006; Alexander et al., 2009
		Output results are generated much more rapidly in comparison to *STRUCTURE*.		
PLINK	A program suite comprising a whole genome association analysis toolset designed to perform a range of basic, large-scale analyses in a computationally efficient manner. It assesses population stratification of whole-genome SNP data through a complete-linkage hierarchical clustering method and it produces a *K* dimensional representation of the substructure through a classical Multidimensional scaling (MDS) technique.	PLINK facilitates the manipulation and analysis of whole-genome data in a computationally efficient way.	Designed for SNP data only (GWAS output).	Purcell et al., 2007; Shi et al., 2011
		It detects and corrects population stratification through identity-by-state and identity-by-descent information.		
		*STRUCTURE* requires additional use of STRAT in a two-stage analysis Using reference GWAS datasets (usually HapMap) PLINK calculates LD values between two SNPs and includes an “LD-clump” analysis that automatically calculates blocks of SNPs in LD across genotyping chip platforms results.		
Multidimensional scaling	Methods such as unrooted neighbor-joining trees, *Principal Component Analysis* (PCA) and *Multidimensional Scaling* (MDS) can also be informative to summarize the genetic similarities and differences between groups of populations. The review of Nassir et al. (2009) suggests that PCA offers computational advantages if the markers are used for controlling population substructure in association studies. The suggested strategy of Nassir et al. (2009) is to use *STRUCTURE* for limiting analyses to particular subject groups, then apply PCA or MDS for association testing. PCA and MDS are also good alternatives to *STRUCTURE* for estimating the number of population clusters. Gao and Starmer (2007) suggest that after using the clustering algorithm implemented in *AW-clust* to identify the major clusters, Bayesian methods can be used to calculate posterior probabilities for individuals from each cluster.	These methods run much more rapidly on large datasets compared to model-based methods.	These methods usually include simple LD correction systems that are not as powerful as those of *STRUCTURE*.	Patterson et al., 2006; Gao and Starmer, 2007; Nassir et al., 2009
		It provides a formal test for the number of significant axes of variation and the presence of population structure in genetic data.	The distance measure and the clustering algorithm can be somewhat arbitrary—i.e., the clustering results may change if another definition of distance or clustering method is applied.	
		It outputs each individual's coordinates along axes of variation instead of trying to classify all individuals into discrete populations, which may not always be the correct model for a particular population history.		
LAMP	LAMP (Local Ancestry in adMixed Populations) estimates locus-specific ancestry (local ancestry) in recently admixed populations through a clustering algorithm that operates on sliding windows of contiguous SNPs. It can also be used to estimate each individual's ancestry (global ancestry).	LAMP is more efficient (about 10^4^ times faster than *STRUCTURE*)—this can be useful when correcting for population stratification in studies of recently admixed populations or with large datasets.	Designed for SNP data only.	Sankararaman et al., 2008; Pasaniuc et al., 2009; Baran et al., 2012
		It does not require the input of genotypes from unadmixed ancestral populations.	It assumes uncorrelated SNPs.	
			It requires the input of several parameters such as global ancestry proportions (that can be calculated with programs such as *STRUCTURE*).	
HAPMIX	A program that uses a haplotype-based method to solve the problem of local ancestry inference in two way admixed population. It uses haplotype information to accurately infer segments of chromosomal ancestry in admixed samples so it particularly useful in association tests for mapping disease genes in recently admixed populations and to do inferences about human history.	HAPMIX makes a more complete use of dense genome-wide data so it produces more accurate results.	Designed for SNP data only.	Price et al., 2009
			It only allows analysis of populations that are the result of mixture between two ancestral populations.	
ADMIXPROGRAM	This method directly evaluates the likelihood function and maximizes it from the hidden Markov Model for admixture mapping using an EM algorithm.	Allows for uncertainty in model parameters, such as the allele frequencies in the parental populations, the number of generations since admixture occurred and the contribution of ancestry at each generation.	Designed for SNP data only.	Zhu et al., 2006

## The step-by-step guide to *STRUCTURE* analysis

The user guide to *STRUCTURE* in Supplementary Material 1, comprises a step-by-step outline and covers the fundamentals of creating an input file and project, the available analysis models, the definition of parameter sets, and how to run a simulation. The guide runs through an example where each of the analysis models and principal parameters of the four software tools are explored. The genotypic data used in this example is available in Table S1. We present suggestions for the analysis and graphical display of the results and optimum estimation of the number of populations detected in a dataset. Additionally, we describe how to handle the parameters included in *CLUMPP*, *distruct* and *STRAT*. *CLUMPP* allows the alignment of different replicates of *STRUCTURE* analysis results from a given number of assumed populations, helping to deal with the commonly encountered problem of multimodality. Clustering algorithms such as the one implemented in *STRUCTURE* can include stochastic simulations during the inferences. This creates a results space composed of different membership coefficients, each one with an associated probability. It is possible that, when analysing the same data set with identical conditions, different final results are obtained. Differences between replicated analysis runs can be of two types: *label change* or *genuine multimodality*. Label change occurs when different replicates create the same membership coefficient estimates but the labels of each group are distinct in each permutation, that is, each cluster does not represent the same predefined population in all runs. It is also possible that the replicates create distinct but likely results that are not equivalent between permutations—genuine multimodality—that is, clusters that represent a particular predefined population have different ancestry membership proportions in each run. This can be the result of difficulties in the search for the possible membership coefficients space or of true biological factors (Jakobsson and Rosenberg, 2007).

Independently of genuine differences between a series of replicated analyses, a method is needed to deal with the replicate results obtained from multiple runs analysing a single dataset. *CLUMPP* software provides three algorithms that identify the best alignment to the replicate results of the cluster analysis. *CLUMPP* reviews the membership coefficient matrices finding those replicates with the best correspondence. Both *STRUCTURE* and *CLUMPP* give similar output files to the extent that *CLUMPP* results can be directly used in standard *STRUCTURE* graphic enhancement software such as *distruct* described below.

An informative way to visualize *STRUCTURE* results is to show each individual as a column segmented into *K* colors representing the estimated membership coefficients. *Distruct* software offers a wide range of options to create images based on the principle of segmented columns and provides visually appealing graphical summaries of the population structure detected in the data. *Distruct* is required by *CLUMPP* but usefully provides an alternative graphical display to the standard *STRUCTURE* bar plots, offering a wider variety of options to create images for much of the *STRUCTURE* output.

Finally, *STRAT* can be used in association studies, enabling the validation of case-control association statistics even in the presence of population structure that can compound the associations suggested by the data.

We focus this work on the 2.3.3 front-end version of *STRUCTURE* but a new version (V2.3.4) was recently released to fix some minor bugs. It is worth noting that the recently released *StrAuto v0.3.1* Python-based software enables an automated approach, albeit from the command line *of Mac* or *Linux* based computers (Chhatre, 2012).

## Concluding remarks

This article presents an updated review of the ubiquitous *STRUCTURE* population analysis software widely applied to a range of population genetics problems. We give recommendations that can guide decisions when analyzing population structure for population genetics and association studies. The review and guide focuses on *STRUCTURE* and the supporting software of *CLUMPP*, *distruct* and *STRAT*. The use of a Bayesian method offers several advantages, especially assigning admixed individuals to population clusters, since it is possible to use prior information to assist the calculation of ancestry proportions for these individuals. Therefore, information on data, the markers applied and the type of analysis desired is relevant before the selection of the analysis parameters.

A simulated example file was thoroughly analyzed and our concluding remark is that there is no one standard analysis parameter in *STRUCTURE*—the data and the study objectives will influence the choice of the most appropriate parameter—and precaution should be used to avoid overestimating the actual population structure present in complex data.

### Conflict of interest statement

The authors declare that the research was conducted in the absence of any commercial or financial relationships that could be construed as a potential conflict of interest.

## Supplementary material

Supplementary Material 1 **A step-by-step guide to *STRUCTURE* and accompanying data processing programs**.

Table S1 **Excel file of genotypes and *STRUCTURE* input data used in the example analyses of Supplementary Material 1**.
