# Supplementary material for: An overview of STRUCTURE: applications, parameter settings, and supporting software
Source: Front Genet. 2013 May 29;4:98. doi: 10.3389/fgene.2013.00098 (PMC3665925; doi:10.3389/fgene.2013.00098)
Supplement: Supplementary Material 1 — A step-by-step guide to STRUCTURE and accompanying data processing programs. [file Presentation1.PDF]

An overview of STRUCTURE: applications, parameter settings and supporting software.

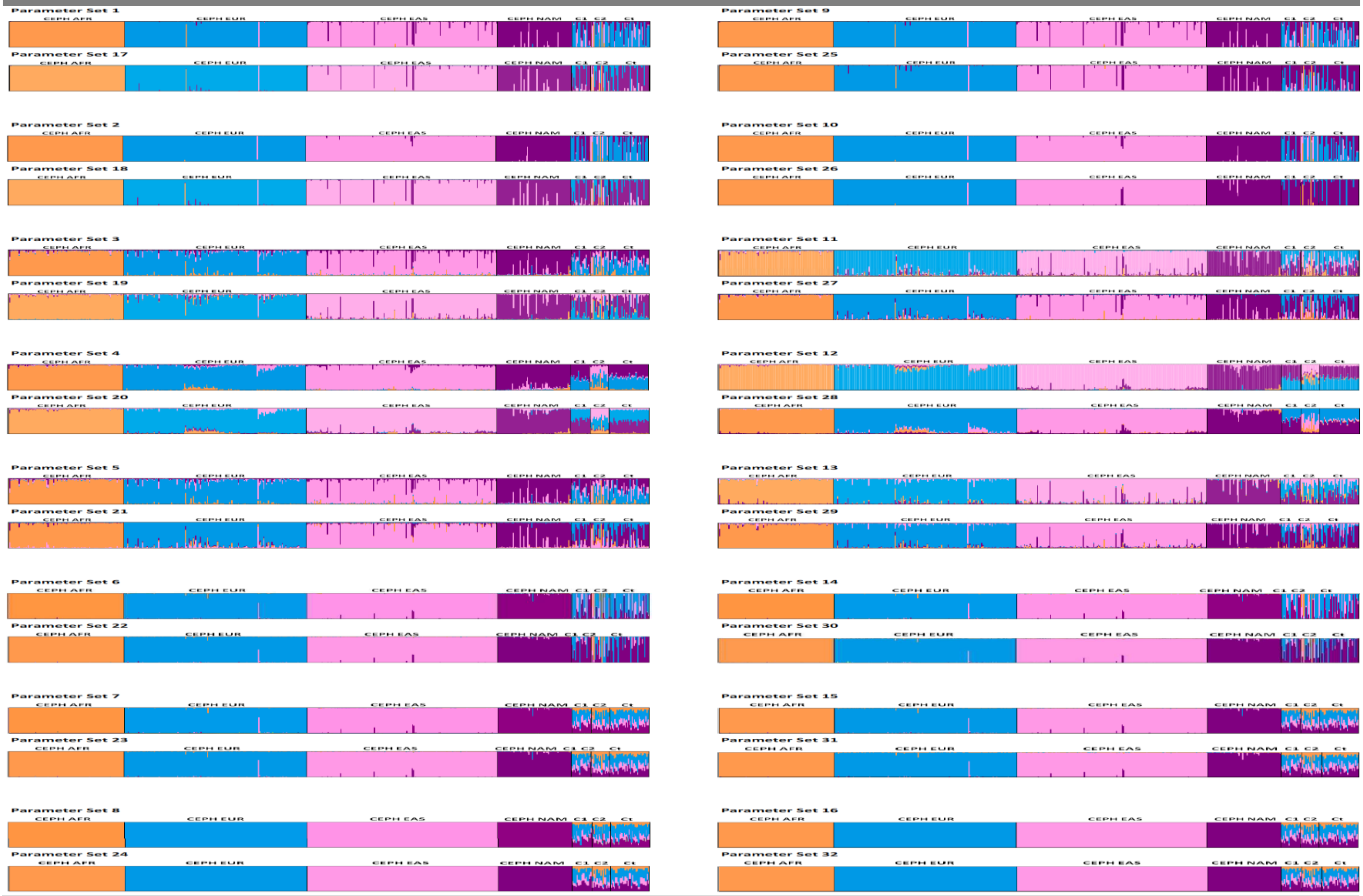

Liliana Porras-Hurtado, Yarimar Ruiz, Carla Santos, Christopher Phillips, Maria Victoria Lareu and Ángel Carracedo

Objectives: We present an up-to-date review of *STRUCTURE* software: one of the most widely used population analysis tools that allows researchers to assess patterns of genetic structure in a set of samples. *STRUCTURE* can identify subsets of the whole sample by detecting allele frequency differences within the data and can assign individuals to those sub-populations based on analysis of likelihoods. The review covers *STRUCTURE*'s most commonly used ancestry and frequency models, plus an overview of the main applications of the software in human genetics including case-control association studies, population genetics and forensic analysis. The review is accompanied by supplementary material providing a step-by-step guide to running *STRUCTURE*.

Methods: With reference to a worked example, we explore the effects of changing the principal analysis parameters on *STRUCTURE* results when analyzing a uniform set of human genetic data. Use of the supporting software: *CLUMPP* and *distruct* is detailed and we provide an overview and worked example of *STRAT* software, applicable to case-control association studies.

Conclusion: The guide offers a simplified view of how *STRUCTURE*, *CLUMPP*, *distruct* and *STRAT* can be applied to provide researchers with an informed choice of parameter settings and supporting software when analyzing their own genetic data.

Supplementary Material 1

A step-by-step guide to *STRUCTURE* and accompanying data processing programs

Table of Contents

1. STRUCTURE VERSION 2.3.3 **3**

1.1 PREPARING A FILE TO RUN IN *STRUCTURE* **3**

a) Rows 3

b) Columns 3

1.2 CREATING A NEW PROJECT IN *STRUCTURE* **4**

1.3 CREATING A NEW ANALYSIS PARAMETER SET **5**

1.4 SELECTING AN ANALYSIS MODEL **6**

a) Ancestry models 6

b) Allele frequency models 9

1.5 RUNNING A SIMULATION **9**

1.6 CHOOSING THE NUMBER OF ITERATIONS **10**

1.7 ANALYSING THE RESULTS **12**

1.8 REPRESENTING THE RESULTS GRAPHICALLY **13**

1.9 ESTIMATING K, THE NUMBER OF POPULATIONS **14**

2. CLUMPP: CLUSTER MATCHING AND PERMUTATION PROGRAM VERSION 1.1.2 **16**

2.1 PARAMETERS INCLUDED IN THE *CLUMPP* ALGORITHMS 16

2.2 RUNNING *CLUMPP* 17

3. DISTRUCT: A PROGRAM FOR THE GRAPHICAL DISPLAY OF POPULATION STRUCTURE VERSION 1.1 **18**

4. STRAT: STRUCTURED POPULATION ASSOCIATION TEST VERSION 1.1 **20**

5. AN APPLIED EXAMPLE **21**

6. DISCUSSION AND FINAL REMARKS **28**

7. REFERENCES **31**

1. *STRUCTURE version 2.3.3 (http://pritch.bsd.uchicago.edu/structure.html (Pritchard et al., 2000a))*

1.1 Preparing a file to run in *STRUCTURE*

Data to be analysed with *STRUCTURE* should be organized in one single matrix (saved as a txt file) where sampled individuals are organized in rows and genetic loci (herein markers) in columns. For diploid organisms the individual data can be organized in two consecutive rows (representing chromosomes) with each locus in one column (option 1, Figure 1) or in one row with each locus in two consecutive columns (option 2, Figure 1). Additional information can be added to the file using a previously established order but this is completely optional.

a) Rows

- **i. Marker name (M_1…M_L)** – optional; as a series of integers or characters; the number of values is the same as the number of markers.
- **ii. Recessive alleles (r_1…r_L)** – only applied to data sets with dominant markers; an integer; generally this row is not included in SNP or STR files (one exception being clinical genetics studies). This label can be used for AFLP data or when the individuals studied are polyploid with ambiguous genotypes.
- **iii. Inter-marker distance (-1…$D_{L-1,L}$)** – optional; a number value applicable to linked markers and representing genetic distances (e.g. centiMorgans) or a measure based on physical distances. It is important that the markers are placed in the same order as their map position. The first marker of each linkage group should be specified by -1 then those following distances are positive values.
- **iv. Phase information ($p_1^{(L)}$…$p_L^{(1)}$)** – optional; only applicable to diploid data; a number value between 0 and 1 used solely with the *linkage model*. This data row should be placed after the genotype data, for each sample. This information is particularly interesting in human studies when haploid data from the X chromosome of males is analysed together with diploid autosomal data.

b) Columns

- **i. Label** – optional; series of integers or characters that identifies each individual. If characters are used some problems may occur when using the post-hoc analysis software: *CLUMPP* and/or *distruct*.
- **ii. Population data** – optional; an integer that represents a population defined by the researcher; by default this information is not used by the grouping algorithm but it can be used to help organize the output file.
- **iii. PopFlag** – optional; 0 or 1 representing the Boolean variable where 1 = TRUE, use population data; and 0 = FALSE, do not use population data.
- **iv. Location** – optional; an integer that represents a sampling location, or other shared characteristic, for each individual. This information is used by the *LOCPRIOR model*. When the location data is the same as the population data it is possible to use the latter value with the LOCPRIOR model without including the location column.

v. **Phenotype** – optional; additional information that represents a phenotype of interest. This information is not used by *STRUCTURE* software but it allows the interaction with *STRAT* in case-control studies.

vi. **Extra columns** – optional; additional information that is ignored by the software but can be useful labels or points of reference for the researcher.

vii. **Genotypic data (Loc 1…Loc L)** – obligatory; an integer; each allele of a determinate marker should be represented by a unique integer (e.g. for SNPs A=1, C=2, G=3, T=4 and for STRs 12.3=123, though a size estimate in nucleotides is commonly used).

The absence of a genotype should be represented by an integer that is not present in the data file (usually -9). In fact, missing alleles do not contribute to the model under Hardy-Weinberg assumptions so they are ignored when found amongst observations (Corander et al., 2003). The same number can be used when there are haploid and diploid data in the same file together with phase information (for example autosomal and X chromosome *loci* in males).

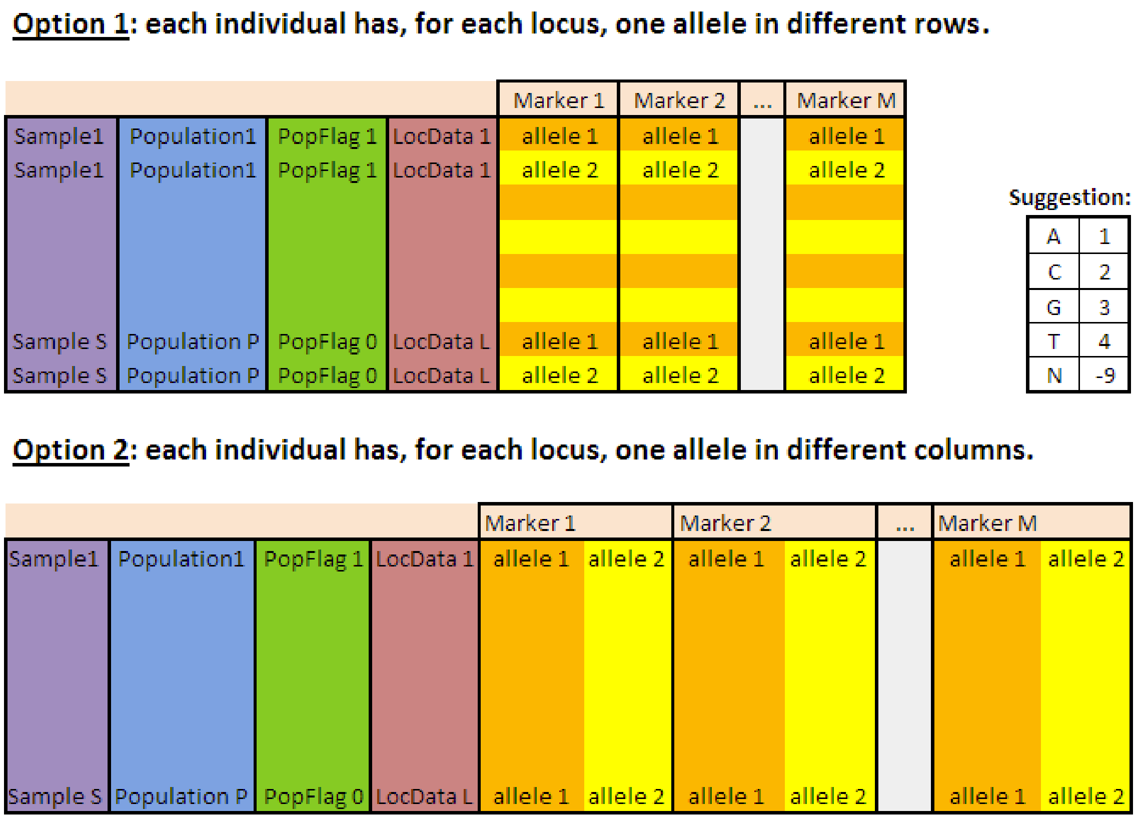

Figure 1: Example of the two possible input files formats to run in *STRUCTURE*. In option 1 the two alleles for each marker are introduced in different rows and in option 2 in different columns. As genotype data is submitted as numerical data a suggestion of conversion in SNP variation is shown.

1.2 Creating a new project in *STRUCTURE*

To create a new project the user selects the option *New project* in the *File* menu of the front-end version of *STRUCTURE*. The first step is to introduce information about the project as: name; output directory where the project and results will be saved and; input file with the genotype data. The second step provides information about the input file: number of individuals; ploidy of the data (by default assumed to be 2 = diploid); number of markers and; the value chosen to represent missing data. It is possible to

verify the number of rows and columns of the input data file in "*Show data file format*" (when using a file of the *Option 1* type shown in Figure 1, there will be one row with M columns that represent the number of markers and 2S rows that corresponds to twice the number of sampled individuals). It is also necessary to indicate the information introduced in rows and columns. By default *STRUCTURE* assumes that the individual data is organized in two consecutive rows with each *locus* in one column. When this is not true (*Option 2*, Figure 1) it is necessary to select the "*special format"* option. It is also important to indicate the number of extra columns when they are present in the input file. Before creating the project file *STRUCTURE* presents a summary of the introduced information. If there are no errors the project will be created and the genotypic data will be visible in the front-end version of *STRUCTURE*.

1.3 Creating a new analysis parameter set

To create a new analysis parameter set the user selects the option *New* in the *Parameter set* menu of the front-end version of *STRUCTURE*.

The first option is to decide how long to run a simulation. Here the user should take into account the *burnin* period (the time that the software runs before it starts to collect the data so as to minimize the effects of the initial configurations) and the time after the *burnin* period when the program runs, to obtain representative estimations of the parameters. Usually a *burnin* period of 10,000-100,000 is sufficient to observe data convergence where key statistical parameters (e.g. α) reach an equilibrium in the values produced. When excessive variation in parameter estimates is observed at the end of the *burnin* period it is necessary to increase the *burnin* time. The degree of variation of parameter values can be checked with the *Data plot* option (in the menu bar of the results window). Graphics such as the example shown in Fig. 2 are obtained and these allow the user to check the degree of variation during the run.

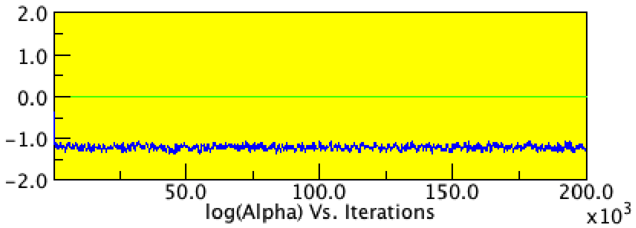

Figure 2: Variation in *alpha* during increasing numbers of iterations in a run. This example used parameter set 3 (see section 5 for details): admixture model, allele frequencies correlated, POPFLAG=1.

To select a suitable run length after the *burnin* it is best to perform several runs for each *K* value, where each run has a different number of MCMC steps, to check if the results obtained are consistent. Generally it is possible to obtain good estimates of the parameters with 10,000-100,000 MCMC steps but to obtain precise estimates of the posterior probability of each *K* value it is often necessary to perform longer runs.

Following the above checks the user should select the ancestry model depending on the study objectives and data to analyse. Different models can be considered: no admixture or admixture models; with or without LOCPRIOR or considering the population information and reference samples; with or without linkage; using the population information to test for migrants. Ancestry models will be described in greater detail later (section 1.4, a).

The user also needs to select an allele frequencies model depending on the study objectives and data to analyse. Two main models can be considered: correlated or

independent allele frequencies. Those two models are described in greater detail in section 1.4, b.

Assessments of *burnin*, number of MCMC repeats, *alpha*, *lambda* or other statistical parameters can now be performed to guide adjustment of analysis factors to more appropriate values. After naming and creating the new parameter set this becomes the active file and a summary of its parameters is presented in the front-end. In addition, a tree with all the defined parameter sets is shown on the left side of the screen.

1.4 Selecting an analysis model

a) Ancestry models

- <u>NO ADMIXTURE MODEL</u> (section 5, Fig. 9, parameter sets 1, 9, 17 and 25). This model assumes that each individual has its origin in only one of the *K* populations and it calculates the posterior probability that the individual has of belonging to population *k*. This model is appropriate to study discrete populations, but as pointed out by Falush *et al.* (2003), this model has the obvious limitation that individuals may have recent ancestors in more than one population.

- <u>ADMIXTURE MODEL</u> (section 5, Fig. 9, parameter sets 3, 11, 19 and 27). Admixture between populations is a common characteristic of real genetic data. This model considers that individuals can have an admixed ancestry such that each individual can inherit a fraction of their genome from ancestors in population *k*. Using this model users will be ignoring possible correlations in ancestry that occur in segments of each chromosome (Falush et al., 2003). The output file includes an approximate median posterior value of the inherited proportions for each individual and population (*individual and population Q-matrices*, see section 1.7).

- <u>LINKAGE MODEL</u> (section 5, Fig. 9, parameter sets 5, 13, 21 and 29). Admixture can create increased linkage disequilibrium between markers that show different allele frequencies in the parental populations. The resulting linkage disequilibrium patterns are largely dependent on the admixture dynamics (Pfaff et al., 2001). The linkage model is a generalization of the admixture model designed to accommodate *admixture linkage disequilibrium* (Falush et al., 2003), i.e., it is able to deal with the correlations that appear between linked markers in recently admixed populations. The admixture model described by Pritchard *et al.* (2000a) considers only the *mixture linkage disequilibrium* (correlations among markers, even unlinked, due to variations in ancestry). Falush *et al.* (2003) incorporated the *admixture linkage disequilibrium model* (additional linkage disequilibrium between linked markers due to correlations in ancestry along each chromosome). However *STRUCTURE* ignores *background linkage disequilibrium*, which usually decays rapidly at a much shorter scale (tens of kilobases in humans) (Falush et al., 2003). The linkage model has the same characteristics as the admixture model but it considers that all the alleles in the same region (linkage group) have an origin in the same ancestral population. This model works better than the original admixture model when linked markers are being used to study admixed populations. The user

should take into consideration that the linkage model was not designed to handle linkage disequilibrium between markers that are very tightly linked. Furthermore this model can only be used when the information about the relative position of the markers (e.g., an accurate genetic map) is available.

- USING PREVIOUS INFORMATION ABOUT THE POPULATIONS: by default *STRUCTURE* only uses genetic information to study population structure. However additional information can be useful and is readily applied to an analysis when using *STRUCTURE* to cluster individuals into groups.

  - **LOCPRIOR models: use of the sampling location as default information to assist clustering (use with sets of data with weak structure signal)** (section 5, Fig. 9, parameter sets 2, 4, 10, 12, 18, 20, 26 and 28). Select the “Use sample location as prior (LOCPRIOR)” option on the Ancestry Model tab under the *no admixture* or *admixture* models. These models allow the correct inference of the population structure and ancestry when the sets of data used have weak structure signals that cannot be detected by basic models available for *STRUCTURE* (Hubisz et al., 2009). LOCPRIOR models assume that individuals from the same sampling location usually belong to the same population, so sampling locations are treated as informative for ancestry. Furthermore, these models have several favourable characteristics: 1) generally they do not find population structure when this is not present; 2) they ignore information about sampling location when individual ancestry is not correlated (Hubisz et al., 2009); 3) when the population structure signals are strong the LOCPRIOR and default models generally provide the same results for the genetic data.

Informativeness of the sampling location data (*r*): this *a posteriori* parameter is useful to detect if the location labels are informative or not for ancestry in the data set tested. When r>>1 the location labels are uninformative about ancestry. But small values of r indicate the ancestry proportions vary substantially amongst locations, i.e. the location information is potentially very informative (Hubisz et al., 2009).

Although this model can be used for data sets with few markers or individuals, it is important to consider the collective informativeness of the study markers before applying this model to assist sample clustering. For example, Bouazake et al. (2009) studied ancestry in ancient DNA with just four ancestry informative genetic markers, but they gave sufficient power to provide sufficient information about individual bio-geographical origin.

The sampling location model is also readily applied when individuals can be classified into discrete groups on the basis of a phenotypic characteristic, ecotype or ethnic group (Hubisz et al., 2009). Under this model the information provided by phenotypes can be used to detect population structure in case-control studies, as outlined later in this guide (section 4). The phenotype must be marked in the corresponding column of the input file. The column “phenotype” contains numeric values (integers) corresponding to the

presence or absence of one (or several) characteristics of interest, i.e. characteristics of case and control samples. This option provides an interface to be used with the *STRAT* post-hoc analysis software.

- **USEPOPINFO model: use of the sampling location to test for migrants or hybrids – only applicable with very informative genetic data** (section 5, Fig. 9, parameter sets 6, 7, 8, 14, 15, 16, 22, 23, 24, 30, 31 and 32)**.** On some occasions the user can observe that the pre-defined groups or sampling locations correspond almost exactly to the groups defined by *STRUCTURE* with the exception of some individuals that seem to be misclassified. This model assumes that the pre-defined populations for each individual (*PopData*) are usually correct. Select “Use population information to test for migrants” option in the Admixture Model tab. It is possible to indicate the number of generations and migration rate and also to choose between the *admixture, no admixture* or *linkage* models.

- **USEPOPINFO model: previous specification of the population of origin of some individuals to help infer the ancestry of individuals with unknown origin** (section 5, Fig. 9, parameter sets 1-16)**.** With this model it is possible to define *learning samples* with pre-defined population-of-origin in particular groups. *STRUCTURE* then attempts to group the remaining individuals. The *learning samples* are defined in the input column *PopFlag* (select “*update allele frequencies using only individuals with POPFLAG=1* data” in the Advanced tab when creating a new parameter set). When *PopFlag*=0, the *PopData* value will be ignored and the ancestry of those individuals will be updated taking into account the admixture or no admixture model, as selected by the user. When there are only a few individuals with no pre-defined population it might be useful to adjust *alpha* (α) by running *STRUCTURE* with a basic model and obtaining an estimated value. Furthermore, the user should run the basic model in the first place to confirm that the pre-defined classification corresponds to real genetic populations. As discussed by Hubisz et al. (2009), the basic models assume *a priori* that all partitions of the *N* individuals into *K* populations are equally likely and that strong statistical evidence should be established to support any particular partition. To avoid confusion with the previous USEPOPINFO model we herein refer to applying USEPOPINFO described in this paragraph as simply *POPFLAG*.

Alpha (α) is a Dirichlet parameter that reflects the relative admixture levels between populations (Hubisz et al., 2009). When $\alpha >> 1$ each individual’s alleles has their origin in all *K* populations in comparable proportions, so the individuals are highly admixed. For values of $\alpha << 1$ each individual has its origin mainly in one population (and each population is equally probable). When α is near 0, the models resemble the *no admixture* model (Falush et al., 2003;Evanno et al., 2005). Using different values of α for each population can help obtain more accurate ancestry estimates since usually not all populations are equally represented in the sample (Falush et al., 2003).

b) Allele frequency models

- INDEPENDENT ALLELE FREQUENCIES MODEL (section 5, Fig. 9, parameters sets 9-16 and 25-32). This model is based on the assumption that allele frequencies for a limited number of markers are not expected to be correlated within a population. Furthermore, ancestral relationships between clusters is not expected (Rosenberg et al., 2005). In this way, allele frequencies in each population are the result of a distribution specified by *lambda* (by default this parameter equals one. The model therefore expects allele frequencies show some differentiation between distinct populations.

- CORRELATED ALLELE FREQUENCIES MODEL (section 5, Fig. 9, parameter sets 1-8 and 17-24). This model uses a multidimensional vector that records the allele frequencies in one hypothetical ancestral population. It is assumed that each of the *K* populations represented in the sample underwent drift from a common ancestral population and that rates of drift varies between populations due to different effective population sizes (Falush et al., 2003). Therefore it can be expected that closely related populations show very similar allele frequencies (Falush et al., 2003) and the alleles in different clusters have correlated frequencies due to shared ancestry (Pritchard et al., 2000a;Rosenberg et al., 2005). For this reason using the correlated allele frequencies model in *STRUCTURE* provides extra flexibility allowing increased power to detect distinct populations even when they are closely related due to recent shared ancestry (Falush et al., 2003;Rosenberg et al., 2005).

  In fact, it is usually the case that the correlated and uncorrelated allele frequencies models produce similar results. Differences are only obtained when there is a high level of correlation across populations (Rosenberg et al., 2005).

Lambda (λ) is the parameter of the distribution of allele frequencies (Evanno et al., 2005) that quantifies the prior allele frequencies. Fixing λ=1 is the appropriate default value for the great majority of data but in certain cases (e.g., SNP data where the minor allele is normally very rare) lower values are more appropriate and work better (Falush et al., 2003). So it is possible to infer λ from an initial basic *STRUCTURE* run and then apply the estimated value on the following simulations as detailed above for alpha.

1.5 Running a simulation

There are two different ways to run a simulation:

a) Run a single *K* value: in the *Parameter Set* menu of the front-end version select *Run*. Define the number of assumed populations (*K*). The software starts the simulation.

b) Create a simulation job: in the *Project* menu of the front-end version of *STRUCTURE* select *Start a Job*. Select the parameter set(s) to analyse and define the number of assumed populations (*K*) and the number of iterations for each *K*. The software starts the simulation and a window with the job progress appears. As described in the FAQ section of the *Google group* forum devoted to *STRUCTURE* (http://groups.google.com/group/structure-software/web/faq) this can result in a stalled

run if the user failed to create a parameter set or to restart *STRUCTURE* after the initial installation. A simple solution is to close and restart the front-end before starting the job, that is, first create the new project and the desired parameter sets, then close the *STRUCTURE* window, restart the software and open the project. Then go directly to the *Project* menu and start a job (it is not necessary to activate a parameter set before starting the job).

A regular point of discussion is the number of possible *K*'s to run. Evanno et al. (2005) advise a suitable range is from *K*=1 or 2 up to the true number of populations plus 3. *Multidimensional Scaling* (MDS) methods such as *Principal Component Analysis* (PCA) are a good alternative to make an estimation of the number of clusters discernable in the genetic data and therefore to use in *STRUCTURE* (Patterson et al., 2006).

1.6 Choosing the number of iterations

A common uncertainty when running a simulation is the number of iterations appropriate for each *K* value since different runs can produce different likelihood values.

To analyze the certainty of the estimates obtained with *STRUCTURE* we performed several replicates of the same analysis. We used the HGDP-CEPH Human Genome Diversity Panel samples from five population groups (Africa, Europe, East Asia, Native America and Oceania) with a set of 59 SNPs originally selected to differentiate those five ancestry components. Firstly we ran five replicates for *K*=2 to *K*=7 with a 200,000 *burnin* period and 200,000 MCMC repeats after *burnin* using the admixture model and considering independent allele frequencies. After calculating the optimum *K* value, which was *K*=5 (data not shown), we performed another 95 iterations of the analysis for *K*=5. We used the values of the major ancestry component when classifying a determinate population to calculate the standard deviation of the median value across iterations, i.e., considering one iteration, two iterations, three iterations, and so on. Those standard deviation values were plotted for each of the five ancestry components (Fig. 3a). The same procedure was followed using the Ln P(K) (Fig. 3b), a measure of the probability of the *K* estimates (see section 1.9). From this analysis we can conclude that when we perform a small number of iterations, the standard deviation associated with each ancestry membership component is higher than when we perform more iterations. We therefore consider it is advisable to do a minimum 20 to 30 iterations to obtain reliable estimates of the ancestry membership proportions of a population.

a)

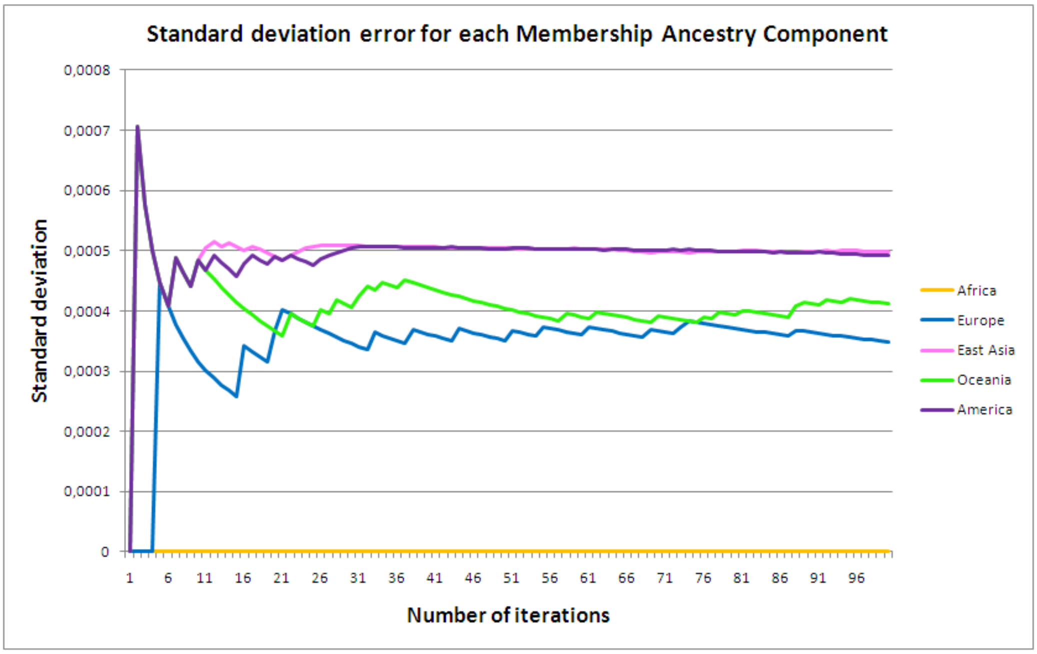

b)

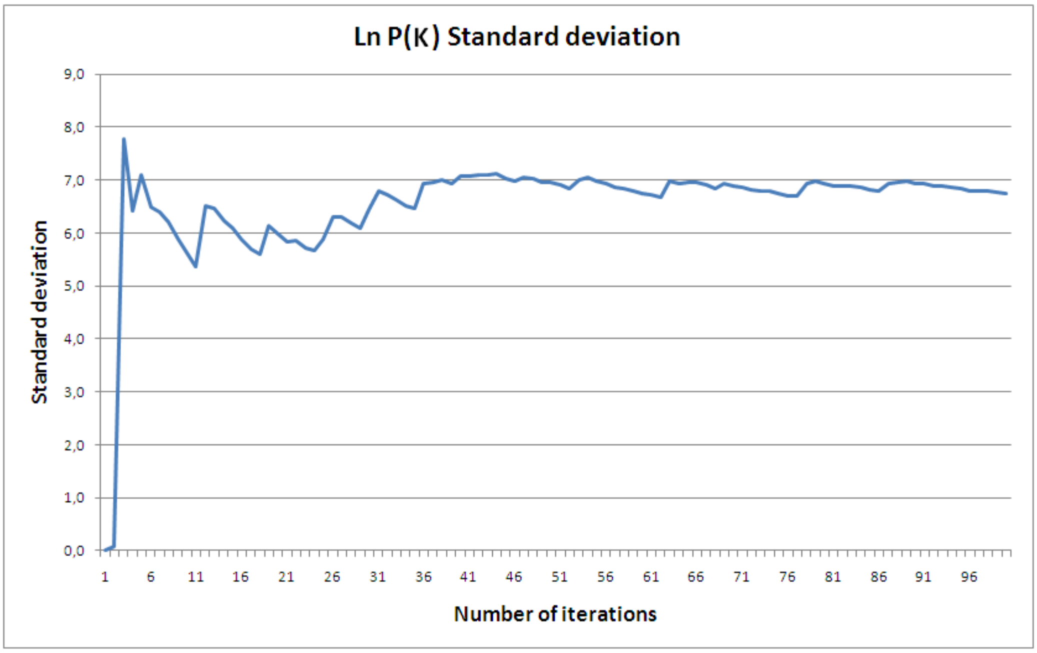

Figure 3: standard deviation variation with the increase of the number of iterations performed. a) variation of the standard deviation associated with the major ancestry membership proportion for each ancestry component (considering a five group comparison); b) variation of the standard deviation associated to the Ln P(K).

1.7 Analysing the results

When the simulations have been completed results files are created (one for each run). They are organized in a tree on the left side of the front-end (Fig. 4a) and when the user selects a file the results are listed.

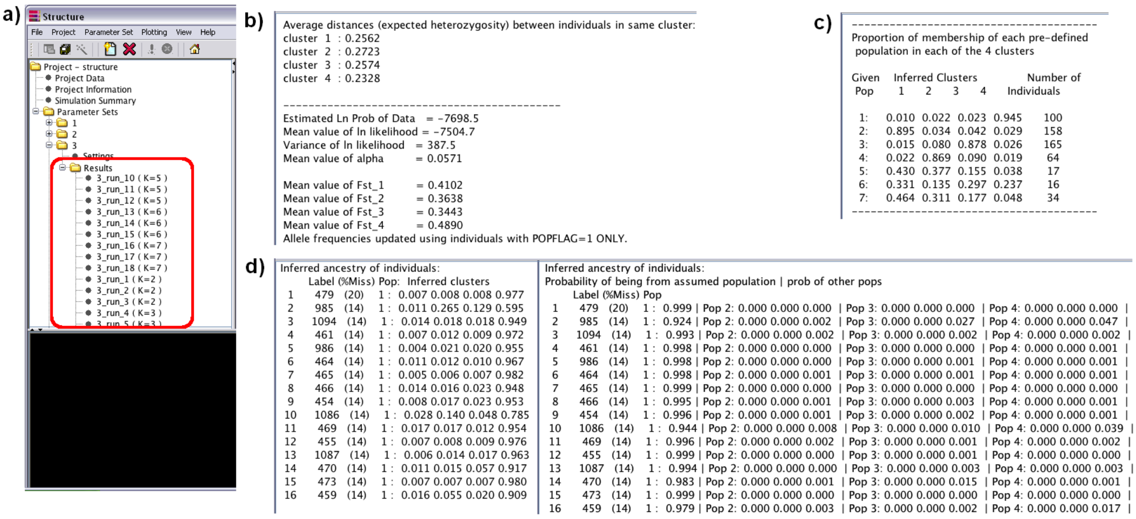

Figure 4: Screen captures of *STRUCTURE* front-end after running simulations. See section 5 for more information about the parameter sets. a) The parameter set tree listing result files; b) example of lnPr (X|K) values and other statistical parameters (parameter set 3, K=4); c) example of a *population Q-matrix* (parameter set 3, K=4); d) beginning section of the *individual Q-matrix* using, left pane: the admixture model (parameter set 3, K=4) and right pane: the POPINFO model (parameter set 7, K=4).

The results file contains several information outputs:

a) Ln Pr (X|K) value This is an estimate of the posterior probability of the simulation (*Estimated Ln Probability of Data* – Fig. 4b). This value is used to estimate the number of populations detected in the sample.

b) *Population Q-matrix* This data set presents the calculated proportions of membership of each pre-defined population in each of the *K* clusters. Considering the example shown in Fig. 4c: seven populations were analysed for *K*=4, where populations 1, 2, 3 and 4 were considered "reference populations" (PopFlag=1) and populations 5, 6 and 7 were "study populations" (PopFlag=0). In this case each cluster will define a particular reference population, that is, each population will have a high ancestry membership proportion in one cluster – cluster 1 defines population 2, cluster 2 defines population 4, cluster 3 defines population 3 and cluster 4 defines population 1. On the other hand, populations 5 and 7 have admixed ancestry with major components of ancestry from populations 2 and 4, and population 6 has admixed ancestry with major components of ancestry from populations 2 and 3. In the example shown, population 5 has the following membership proportions in each cluster: 0.430, 0.377, 0.155, 0.038. So population 5 represents an admixed population with 43.0% of ancestry from population 2, 37.7% of ancestry from population 4, 15.5% from population 3 and 3.8% from population 1.

c) *Individual Q-matrix* This data listing presents the inferred ancestry components of each individual in each of the *K* clusters when using the admixture model or the

membership probability when using the USEPOPINFO model (Fig. 4d). Interpretation of this data is similar to that of the *population Q-matrix* but using the admixture model it is possible to estimate the ancestry membership proportions of each individual. When using the POPINFO model the individual results are presented as the probability of originating from the assumed population versus the probability of having origins from each of the other populations. In this case asterisks after the probabilities indicate misclassified individuals.

Other population genetic information including F_{ST} values and allele frequencies are presented.

1.8 Representing the results graphically

The estimated membership coefficients of the analysed individuals in each group can be represented in two different types of plots:

a) **Bar plot** Each individual of the sample is represented by a vertical line divided into *K* coloured segments with the length of each segment being proportional to the estimated membership in each of the inferred *K* groups (Fig. 5a). This graphic can represent the data set as a set of population groups or more detail can be obtained by opting for individual sample information. If the *plot in multiple lines option* is selected it is possible to observe individual bar plot representations. To access this graphic select *Bar plot* in the menu bar of the results window in the front-end version.

b) **Triangle plot** Each individual is represented as a coloured point in one or a series of triangles (Fig. 5b). Each colour corresponds to the population label associated in the input data file. The estimated ancestry vector for each individual consists of *K* components that sum to one. Proximity to any one triangle vertex corresponds to a high membership value for the population denoted by the vertex so this type of graphic is particularly useful to represent *K*=3 data since ancestry vectors can be represented in a single plot where each of the three components is given by the distance to each of the triangle vertices. When there are K>3 clusters, populations are combined to form a single vertex (as shown for populations 2, 4-7 for K=4 in Fig. 5b). Therefore, although triangle plots allow straightforward visualization of the data for *K*=3, for *K*>3 bar plots are generally easier to read. To access this graphic select *Triangle plot* in the menu bar of the results window in the front-end version.

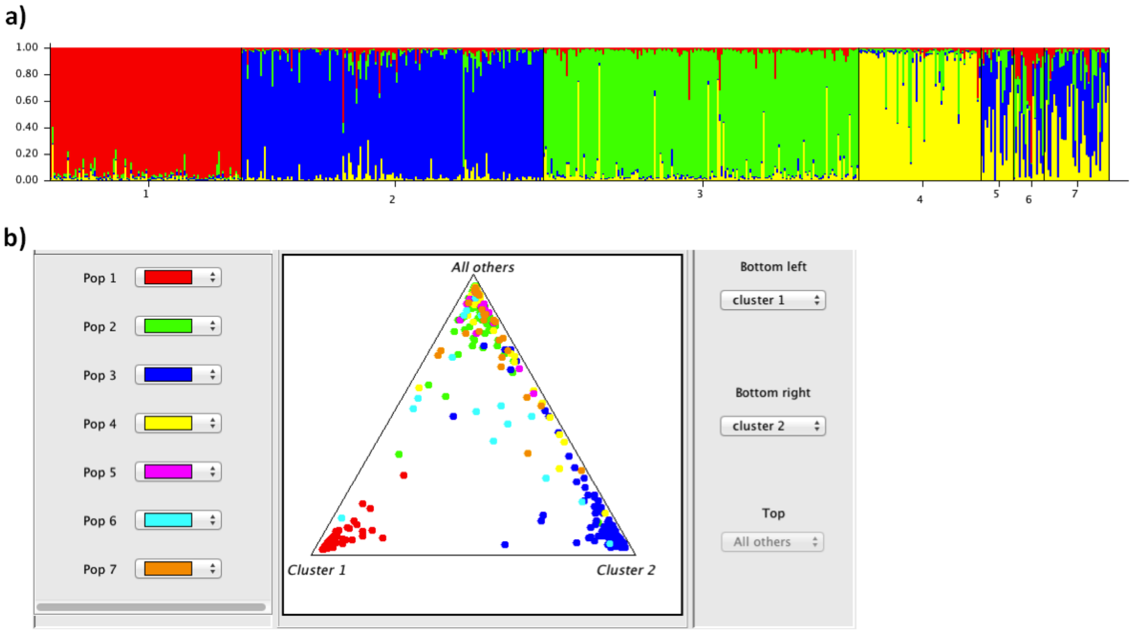

Figure 5: Analysis parameters: Parameter Set 3 (see section 5 for details) - admixture model, allele frequencies correlated, POPFLAG=1. a) bar plot representation for *K*=4. b) triangle plot representation for *K*=4.

c) Statistical parameters plots In the menu bar of the results window in the front-end version it is also possible to select *Data plot* or *Histogram*. Those graphics present some of the statistical parameters calculated throughout the simulation that can be useful when assessing the quality of the estimates obtained from the run.

1.9 Estimating K, the number of populations

This aspect of *STRUCTURE* analysis requires careful consideration as it is difficult to obtain a precise estimate of Pr(X|K), since the estimated *K* value can often depend on the model used to analyse the data (Falush et al., 2003). The method implemented in *STRUCTURE* only permits an *ad hoc* approximation, i.e., with a particular purpose. Furthermore, the biological interpretation of *K* might not be straightforward. So the best strategy is to focus the analysis of a *K* value that captures the majority of the structure present in the data and that can be inferred to be biologically reasonable. As pointed out by Rosenberg et al. (2005) several factors can influence clustering, including: the number of markers typed; sample sizes; the number of clusters and; allele frequency correlations.

a) Estimating K. To estimate *K* it is necessary to allow α to vary during the simulations and to run *STRUCTURE* for different *K* values. For each simulation a posterior probability value ("*Estimated Ln Prob of Data*" or lnPr(X|K)) is calculated. The user needs to verify if these estimates are consistent between different runs so it is advisable to perform several independent simulations for each *K* value. With these values it is possible to calculate the posterior probability for each *K* (Pritchard et al., 2000a):

$$Posterior\ Prob\ K1 = \frac{e^{Ln\ \Pr(X|K1)}}{e^{Ln\ \Pr(X|K1)} + e^{Ln\ \Pr(X|K2)} + \ldots + e^{Ln\ \Pr(X|Kmax)}}$$

When there is real population structure it is possible to find linkage disequilibrium between unlinked markers that will lead to deviations in the Hardy-Weinberg

proportions, but the existence of endogamy or genotyping errors (e.g., undetected null-alleles) can have the same effect.

b) Selecting the appropriate *K* value from the data. Usually Pr(K) values are small for estimates of *K* below the appropriate estimate of populations, while Pr(K) values tend to stabilize with bigger *K* values. Thus Pr(K) values that follow from the most appropriate value create a plateau of values. Evanno et al. (2005) describe a method to estimate *K* based on the second order rate of change of the likelihood function with respect to *K*. Their method was implemented in the program *Structure Harvester* (Earl and vonHoldt, 2012) that calculates *delta K* and plots the median value of lnPr(K) for each *K* (Fig. 6). The only requirements are the use of at least three sequential *K* values and the same number of replicates (>1) for all *K* values. *Delta K* should be used together with the logarithm probability of *K*, alpha value and the individual assignment patterns (Evanno et al., 2005).

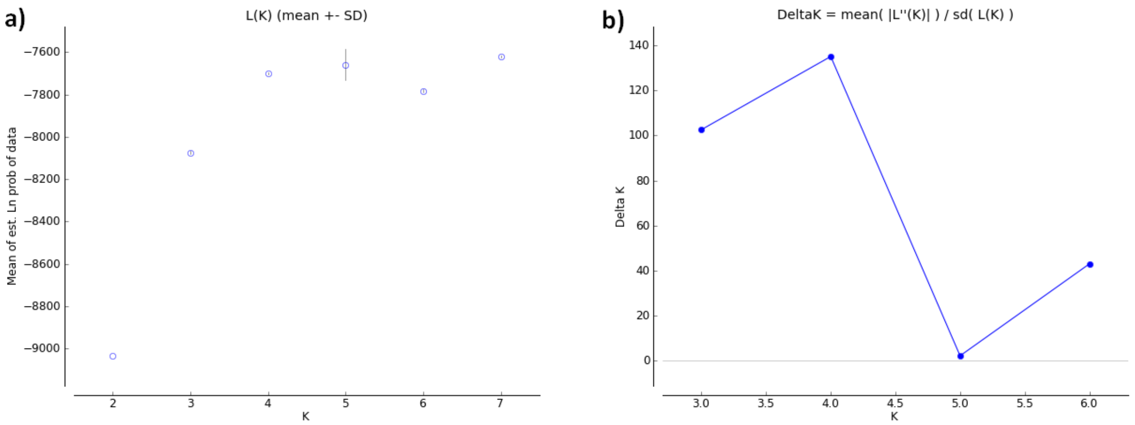

Figure 6: Graphic representation of the estimated probability of data for each *K* value. Analysis parameters: Parameter Set 3 (see section 5 for details) - admixture model, allele frequencies correlated, POPFLAG=1, with three replicates for each K value. a) the median and variance of the estimated probability value for each *K* value. It is possible to observe a plateau after *K*=4. b) *delta K* calculated by the Evanno *et al.* (2005) method. The maximum value is observed at *K*=4.

When several *K* values have similar Ln Pr(X|K) estimates it can be inferred with reasonable certainty that the smallest is the most appropriate for the data (in Fig. 6a this is *K*=4). Although it is not always possible to know the real *K* value the user should always select the lowest value of *K* that captures the maximum degree of structure detected in the data. Furthermore when there is no population structure the proportion of each individual assigned to each population is roughly symmetrical (~1/*K* in each population) and the individuals are equally admixed. If some of the individuals are strongly assigned to one population or if the proportions assigned to each group are asymmetrical there is strong evidence that real population structure exists.

In summary, the user should be guarded about population structure inferred from very small differences of Pr(K) and when there is no clear biological interpretation to assignments of *K*. Likewise when there is approximately symmetrical membership proportions between groups and individuals are not clearly assigned to any one group it is very likely that no population structure is present in the sample.

2. *CLUMPP: CLUster Matching and Permutation Program version 1.1.2 (http://www.stanford.edu/group/rosenberglab/clumpp.html, (Jakobsson and Rosenberg, 2007))*

Because replicating *STRUCTURE* runs creates stochastic effects it is particularly important to simplify the assessment of replicate data by calculating medians. *CLUMPP* analyzes the results from replicated *STRUCTURE* runs by collating all the data into a matrix (the *Q-matrix*) of individual membership co-efficients and population ancestry components.

2.1 Parameters included in the *CLUMPP* algorithms

K is an integer that represents the number of groups.

C is an integer that represents the number of populations or individuals (individual matrix – DATATYPE=0; population matrix – DATATYPE=1) being studied.

R is an integer that represents the number of Q matrices or runs that are aligned.

A *Q-matrix* is a ***C*** x ***K*** membership coefficient matrix resulting from the analysis of a *K* value (number of assumed populations in *STRUCTURE*) with *C* rows corresponding to the numbers of individuals or populations and *K* columns corresponding to distinct assumed population groups. *CLUMPP* tries to maximize the similarity measure: ***G*** between Q matrices from *R* replicates. Using the *G* values it is possible to calculate the median similarity value through pair comparison: ***H***. A second function of similarity, ***G'***, is standardized to vary between 0 and 1. A median similarity value is calculated from *G'* denoted by: ***H'***.

M is an integer that represents the algorithm used to align the different runs. Possible values are: 1 (*FullSearch* algorithm), 2 (*Greedy* algorithm) and 3 (*LargeKGreedy* algorithm). *CLUMPP* uses these three algorithms to search the optimum alignment of the *R* replicates. In decreasing order of the scope of the search made and in increasing order of computational speed the algorithms are:

- *FullSearch* – this calculates *H* to all possible alignments of the *K* groups in the *R* replicates. Considering all possible permutation vectors the *FullSearch* algorithm calculates the median similarity value and returns the permutation vector that maximizes the symmetric similarity coefficient. Despite having a slow computation speed it guarantees the optimum alignment of groups in the multiple runs will be found.
- *Greedy* – this calculates all possible permutations for each run but the number of *G* computations performed for each permutation is *r*-1 (*r* is between 2 and *R*). As the order in which the runs are considered can affect the results the user should perform different run sequences. *CLUMPP* offers three distinct options to test different sequences: to test all the sequences, to test a predefined number of random sequences and to test a specific set of sequences defined by the user.
- *LargeKGreedy* – when *K*>15 the number of permutations is very large and it becomes impractical to calculate *G* for all permutations of a particular pair of Q matrices. So a proportion of the possible permutations are tested. As with the *Greedy* algorithm, the order in which runs are considered can affect the results and the same three options to select the sequence of runs are available.

W is a *Boolean* type variable that it is used when the data comes from populations with different number of individuals (as indicated in the last column of the *popfile* data file). It is possible to calculate *H* (or *H'*) taking into account the "weight" of the number of individuals in each population. As it is a *Boolean* type variable, *W* can assume the values: 0 when the number of individuals in each population is not used, i.e., all populations have the same weight or 1 when the alignment takes into account the number of individuals in each population. This information is only relevant when DATATYPE=1, i.e., when population data is being analysed. When DATATYPE=0 this option (*W*) is automatically inactivated.

S is an integer that represents the pairwise similarity statistic. Possible values of S are 1 for the *G* statistic and 2 for the *G'* statistic.

The authors usually opt to use *M*=1, *W*=0 and *S*=2, but for more information about the possible combinations between algorithms plus the *GREEDY_OPTION* and its use we recommend further review of the software manual.

2.2 Running *CLUMPP*

CLUMPP uses the *STRUCTURE* output Q matrices. Files should be opened with a text editor and it is very important to keep the file extensions intact otherwise *CLUMPP* will not then recognize them.

The first step is to create the input files by copying the population Q matrices from the results files of each *STRUCTURE* replicate for the same *K* value and pasting them into the *popfile* file, leaving a blank line between each matrix. This operation is repeated with the individual Q matrices by pasting them into the *indfile* file. *Structure Harvester* output provides ready-to-use *indfile* and *popfile* files from each K value for *CLUMPP*.

Next the parameters of analysis need to be defined. In the *paramfile* modify *DATATYPE*, *K*, *C*, *R* and *M* (in most cases we recommend the *FullSearch* algorithm) accordingly to the data. A separate run should be made to obtain the population and the individual permutated matrices. So the user should define DATATYPE=0 and C=number of individuals when creating the individual permutated *Q-matrix*. In the case of the population permutated *Q-matrix*, DATATYPE should be equal to 1 and C to the number of populations. To run the program the user selects the executable file. The permutated table will be created in the *outfile* file. The user should copy the population table to a new file before initiating the second run to obtain the individual table or change the OUTFILE name in the *paramfile*. Take into account that the individual's label in *STRUCTURE* is an alphanumerical code but *CLUMPP* will report an error when trying to execute the program to create the individual permutated *Q-matrix*. So we advise the removal of the letters from any labels (this is automatically done by *Structure Harvester*). The resulting tables are then used by *distruct* to generate the bar plots.

3. *Distruct: a program for the graphical display of population structure version 1.1 (http://www.stanford.edu/group/rosenberglab/distruct.html (Jakobsson and Rosenberg, 2007))*

To run *distruct* the user can apply the results from *STRUCTURE* or from *CLUMPP* analyses obtained as described above. As with *CLUMPP*, files should be open with a text editor and due regard made not to change the file extension.

The first step is to create the input files by copying the *population Q-matrix* from the result file of *STRUCTURE* or *CLUMPP* and pasting into the *popq* file. This process is repeated with the *individual Q-matrix* pasted into the *indivq* file. In the files *names* and *languages* the names to be printed respectively below and above the graphic are defined – in separate rows write the numeric code that corresponds to each population followed by a space and the desired name. In the file *perm* it is possible to define the colour associated to each population group. The user should indicate the same number of colours as *K* assumed populations, in different rows writing the number that identifies each results group (each column of the *population Q-matrix*) followed by the code that identifies the desired colour. A complete list of colours and their codes is available in the software help file. Finally, in the *drawparams* file it is possible to define the analysis parameters. There are several parameters that can be changed but the most important for the program to run are *K* (number of assumed populations), *NUMPOPS* (number of populations) and *NUMINDS* (number of individuals). All other parameters define graphical characteristics: letter size, distance between the text and the graphic, height of the graphic, thickness of the columns that represent individuals, thickness of the outline, vertical/horizontal orientation, and other factors. After each parameter the symbol "//" indicates that the following text are commentaries that will not be used by the program and explaining each of the parameters.

In the *distruct* folder there are two files that allow the program to run: an executable file and an *MS-DOS* file. The difference between them is that the executable file automatically closes at the end of the computations while the *MS-DOS* file opens a command line window that remains open and where some results are presented. Opting for the *MS-DOS* file can be particularly useful since it allows the user to see if errors have occurred during the run. When *distruct* finishes the run the *PS* file will be changed. That file can be opened with dedicated programs such as *GhostView* to view the results (example in Fig. 7).

STRUCTURE

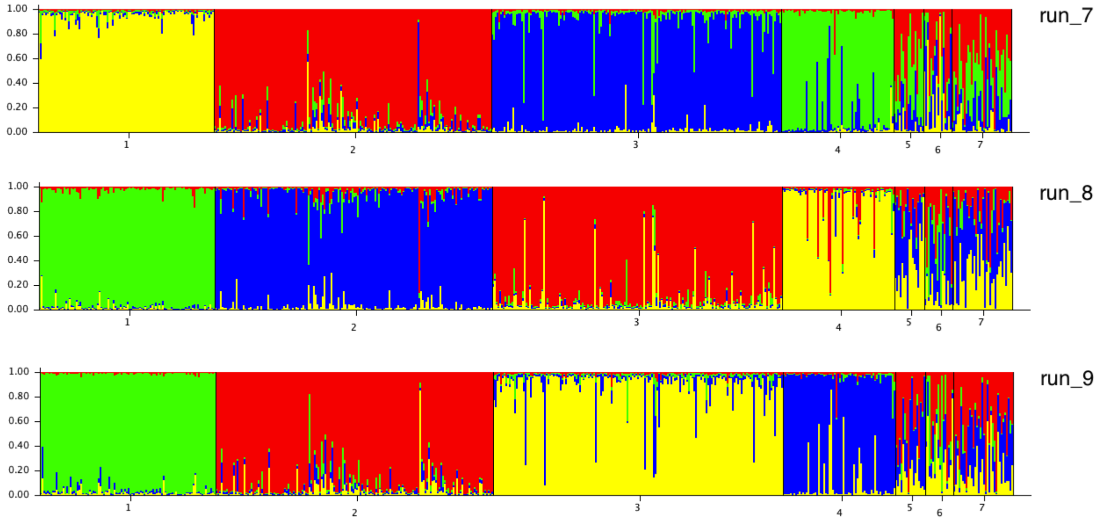

DISTRUCT

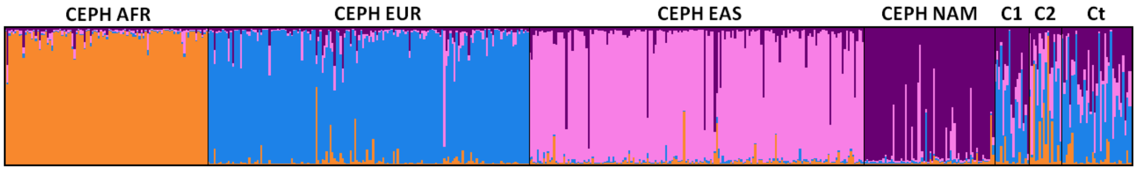

Figure 7: Estimated membership proportions represented in bar plots obtained directly with *STRUCTURE* (top) or processed with *CLUMPP* and *distruct* (bottom). Analysis parameters: Parameter Set 3 (see section 5 for details) - admixture model, allele frequencies correlated, POPFLAG=1, K=4. Note the existence of label change multimodality between the different replicates in *STRUCTURE* (for more information about multimodality see the main article).

4. *STRAT: STRuctured population Association Test version 1.1* (http://pritch.bsd.uchicago.edu/software/STRAT.html (Pritchard et al., 2000b))

When analyzing case-control studies it is important to check that no substructure exists as this could lead to spurious associations, the most common source of false signals of association, especially when the sample contains two populations. The program *STRAT* is designed to apply *STRUCTURE* analysis to test for presence of substructure in case-control sample groups.

To use *STRAT* a version of *STRUCTURE* should be installed (or the result folder from a *STRUCTURE* analysis) along with the *STRAT* program.

The following guidelines provide the steps to run *STRAT*:

1) Run *STRUCTURE* following the previous suggested steps. It is important to verify that the option *PrintQhat* (in the *Advanced* tab), which specifies the essential data to run *STRAT,* is selected when creating a new parameter set for the analysis.
2) *STRUCTURE* implements two models of allele frequencies: the default model assumes that the underlying allele frequencies in different populations are independent (and thus usually quite different). A second model assumes that the allele frequencies are all close to the mean frequency in the sample (for this model set *FREQSCORR*=1 in the *extraparams* file). For human data sets in which the populations are from different continents the first model works well. However, when considering closely related populations (e.g. Chinese and Japanese), *STRUCTURE* does not perform well under the default model, often grouping all the individuals into one population. However applying the model with *FREQSCORR*=1 produces accurate assignments. Also, in such cases, if there is little admixture, the model without admixture (*NOADMIX*=1) tends to produce better results.
3) Copy the *project_data* file from the *STRUCTURE* results directory to the *STRAT* folder – this is the *INPUT* file that contains the genotypes of both case and control samples and includes additional information on ancestry. A column with numerical data specifying which samples correspond to cases and controls can also be added. These case-control samples may have one or more phenotypic variables that are the object of study.
4) The output *q*-file from *STRUCTURE* is used as a second input file in *STRAT*. The text files *mainparams* and *extraparams* that are the same as the *STRUCTURE* results are also necessary for *STRAT* to run so they should be copied to the *STRAT* folder.
5) The two input files should be indicated in the *mainparams* file – the *project_data* in the *define INPUT* entry and the *STRUCTURE* results file (*xxx_run_1*) in the *define OUTPUT* entry. Using these two files it is possible to start running *STRAT*.
6) It is better to run *STRAT* from the command line: change the directory (cd + pathway to *STRAT* folder + *Enter*) and then execute the software (type *STRAT* and *Enter*).
7) The software outputs, in the command line window and in the output file *xxx_run_1_p* (in the *STRUCTURE* results folder) form lines of data as shown by the following example:

   63: chisq= 5.796 1 df; TS = 0.41, p = 6.84000e-01.

This line gives the locus-number in the input file (63), the value of the chi-square test of association assuming no population structure (5.796), the number of degrees of freedom (1), the value of the *STRAT* test-statistic (0.41), and the *STRAT* *p*-value (6.84000e-01). The *p*-value is represented in scientific notation: the *p*-value here is 6.84×10^{-1}. Asterisks are printed at the end of the line for small *p*-values. Then *STRAT* will produce a table of expected probabilities of the simulated *p*-values under the assumption of no population structure. The output test statistic values are calculated as suggested by Pritchard and Rosenberg (1999) (Pritchard and Rosenberg, 1999) for testing whether the case and control samples are mismatched and hence population structure is a possible compounding factor.

8) A summary of the estimated allele frequencies is printed into the output file *xxx_run_1_fr*. It shows the allele frequencies for biallelic markers, assuming two populations and two phenotypes.

5. An applied example

An example file (Supplementary Table 1) was created with 487 individuals (100 Africans: CEPH AFR, 158 Europeans: CEPH EUR, 165 East Asians: CEPH EAS, and 64 Native Americans: CEPH NAM) from the HGDP-CEPH panel of samples. An artificial case-control group was created using Mexican and Puerto Rican samples from HapMap: in total 67 samples divided in Cases 1 (C1), Cases 2 (C2) and Controls (Ct). Different SNP markers were used: 9 AIM-SNPs (two of them triallelic), 3 phenotype associated SNPs and 5 AIM-SNPs on the X-chromosome. The phenotype and the X-SNPs are linked forming two distinct linkage disequilibrium groups – their genetic distance was used to define linkage disequilibrium groups.

As an initial analysis of the data, a PCA was generated using a homemade script in *R* v2.13.1 (Fig. 8). The first principal component explains 27.77% of the information contained in the data set and the second principal component explains 20.20%. It is possible to observe that the HGDP-CEPH individuals can be grouped into four main groups, as would be expected from their known origins and that Case 1 samples present a greater overlap with the Control samples than the Case 2 samples.

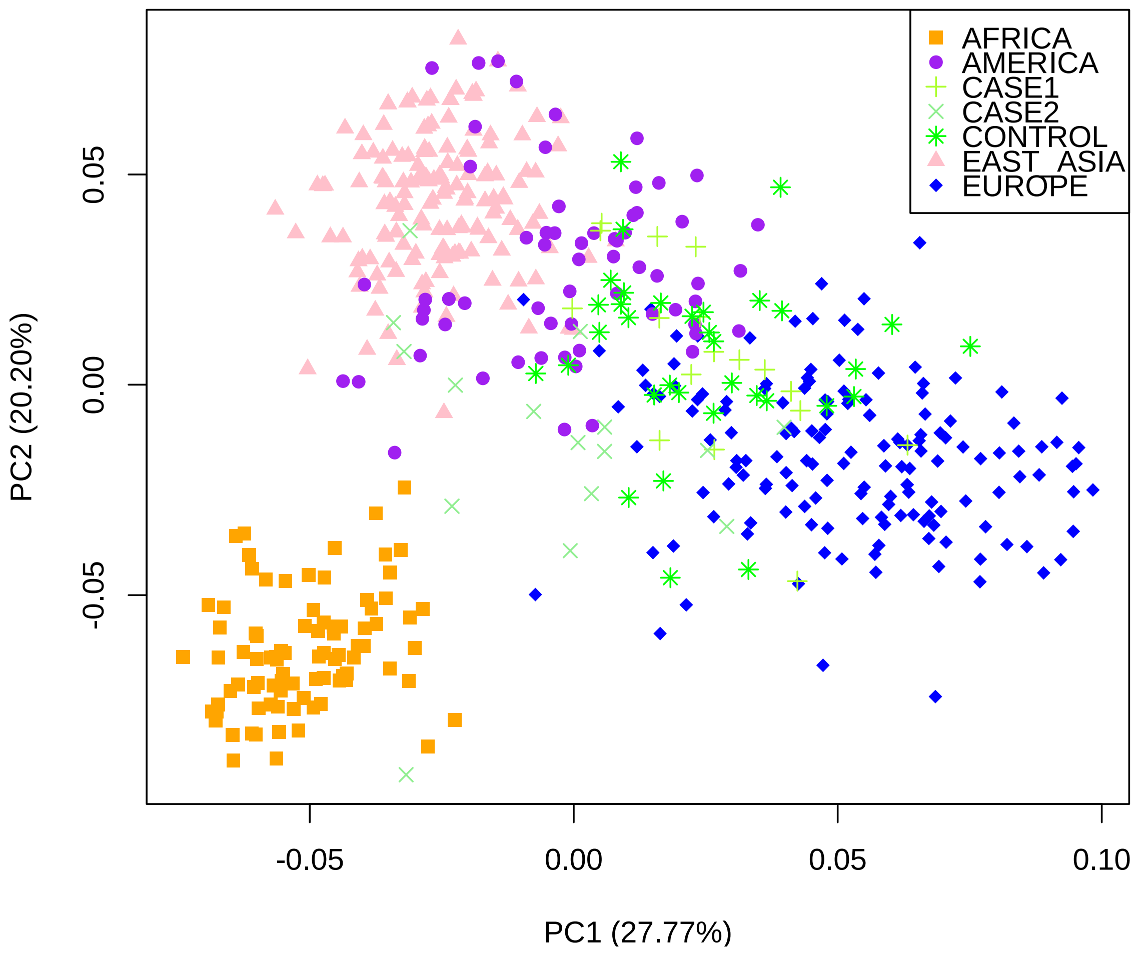

Figure 8: Principal Component Analysis plot of the samples under study.

A large number of parameter combinations were used to analyse the samples in *STRUCTURE* (Table 1). Each parameter set was analysed with three replicates for *K*=2 to *K*=7 and all runs were performed with 100,000 *burnin* period and 100,000 MCMC repeats after *burnin*. The optimum *K* value was assessed through the analysis of the Ln P(D) distribution plots and *delta K* values from *Structure Harvester* (Earl and vonHoldt, 2012). Bar plots of *K*=4 were constructed with *CLUMPP*, to align the three replicates, and *distruct*. Special care should be taken with the parameter sets using POPINFO ancestry models, as the *individual Q-matrix* represents estimated probabilities (and not ancestry membership proportions) the table does not have a format suitable to use with *CLUMPP* and *distruct*. From the above analyses *K*=4 bar plots for parameter sets 6-8, 14-16, 22-24 and 30-32 were obtained directly from *STRUCTURE*. The bar plot corresponding to the *K*=4 replicate with an intermediate value of estimated Ln P(D) was used and image editing was applied to match colours between plots. Complete results from these simulations can be found in Figure 9.

Table 1: Parameter set used in different *STRUCTURE* simulations.

***STRUCTURE* simulations**			
**Parameter Set**	**PopFlag**	**Allele Frequencies Model**	**Ancestry Model**
**1**	On	Correlated	No Admixture
**2**	On	Correlated	No Admixture LOCPRIOR
**3**	On	Correlated	Admixture
**4**	On	Correlated	Admixture LOCPRIOR
**5**	On	Correlated	Linkage
**6**	On	Correlated	POPINFO No Admixture
**7**	On	Correlated	POPINFO Admixture
**8**	On	Correlated	POPINFO Linkage
**9**	On	Independent	No Admixture
**10**	On	Independent	No Admixture LOCPRIOR
**11**	On	Independent	Admixture
**12**	On	Independent	Admixture LOCPRIOR
**13**	On	Independent	Linkage
**14**	On	Independent	POPINFO No Admixture
**15**	On	Independent	POPINFO Admixture
**16**	On	Independent	POPINFO Linkage
**17**	Off	Correlated	No Admixture
**18**	Off	Correlated	No Admixture LOCPRIOR
**19**	Off	Correlated	Admixture
**20**	Off	Correlated	Admixture LOCPRIOR
**21**	Off	Correlated	Linkage
**22**	Off	Correlated	POPINFO No Admixture
**23**	Off	Correlated	POPINFO Admixture
**24**	Off	Correlated	POPINFO Linkage
**25**	Off	Independent	No Admixture
**26**	Off	Independent	No Admixture LOCPRIOR
**27**	Off	Independent	Admixture
**28**	Off	Independent	Admixture LOCPRIOR
**29**	Off	Independent	Linkage
**30**	Off	Independent	POPINFO No Admixture
**31**	Off	Independent	POPINFO Admixture
**32**	Off	Independent	POPINFO Linkage

Figure 9 First page: plots with the Ln P(D) median values for each *K* are presented to estimate the optimum number of populations under each of the simulated parameter sets (see Table 1). Second page, bar plots representing *K*=4 for each parameter set are presented. These were obtained with *CLUMPP*, to align the three replicates for *K*=4 (all runs were performed with 100,000 *burnin* period and 100,000 MCMC repeats after *burnin*), and *distruct*. The exception were the POPINFO parameter sets for which direct *STRUCTURE* bar plot outputs were used (*K*=4 replicate with intermediate Ln P(D) value) together with image editing to match colours.

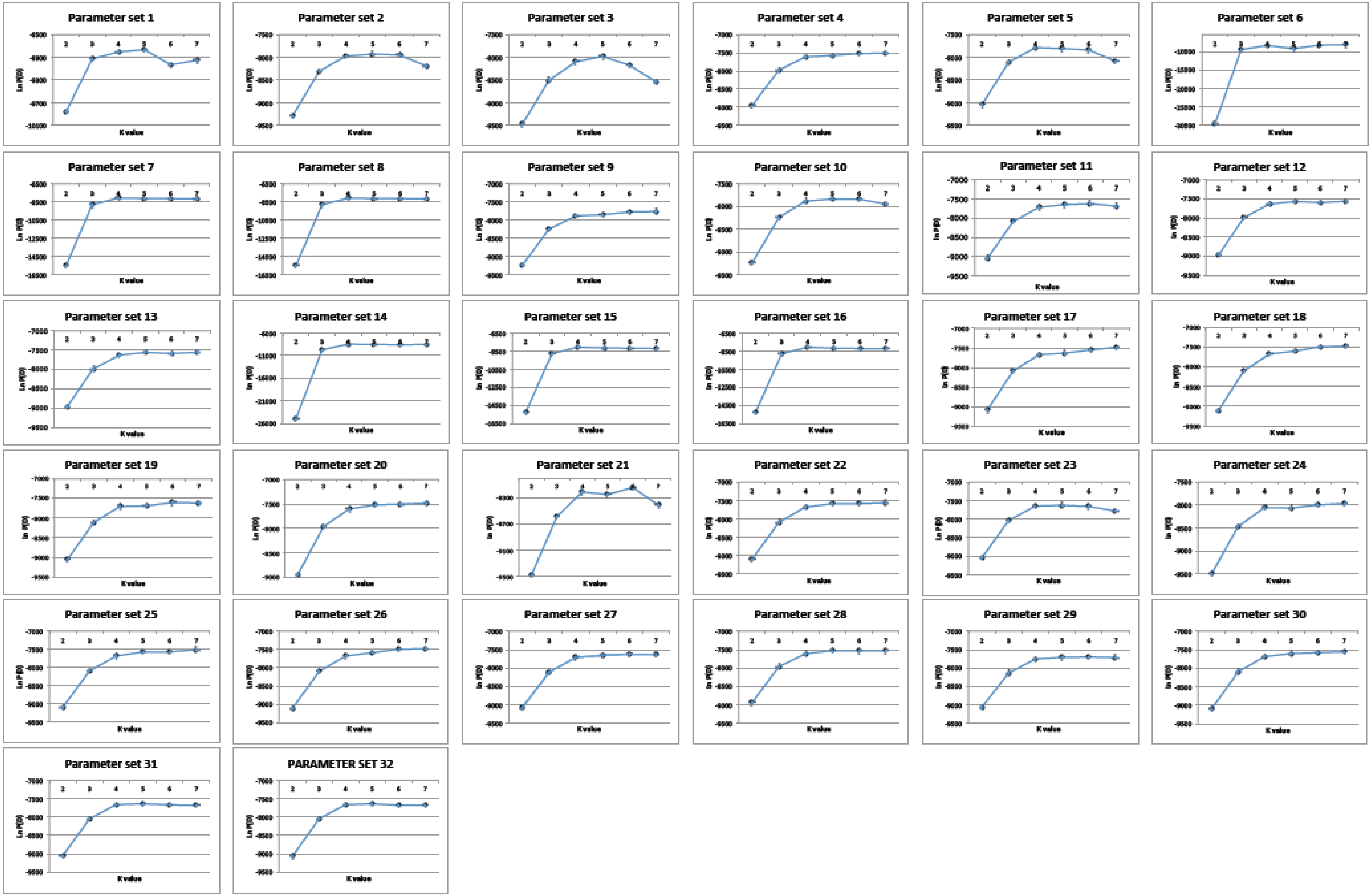
Parameter set 1
Parameter set 2
Parameter set 3
Parameter set 4
Parameter set 5
Parameter set 6
Parameter set 7
Parameter set 8
Parameter set 9
Parameter set 10
Parameter set 11
Parameter set 12
Parameter set 13
Parameter set 14
Parameter set 15
Parameter set 16
Parameter set 17
Parameter set 18
Parameter set 19
Parameter set 20
Parameter set 21
Parameter set 22
Parameter set 23
Parameter set 24
Parameter set 25
Parameter set 26
Parameter set 27
Parameter set 28
Parameter set 29
Parameter set 30
Parameter set 31
PARAMETER SET 32

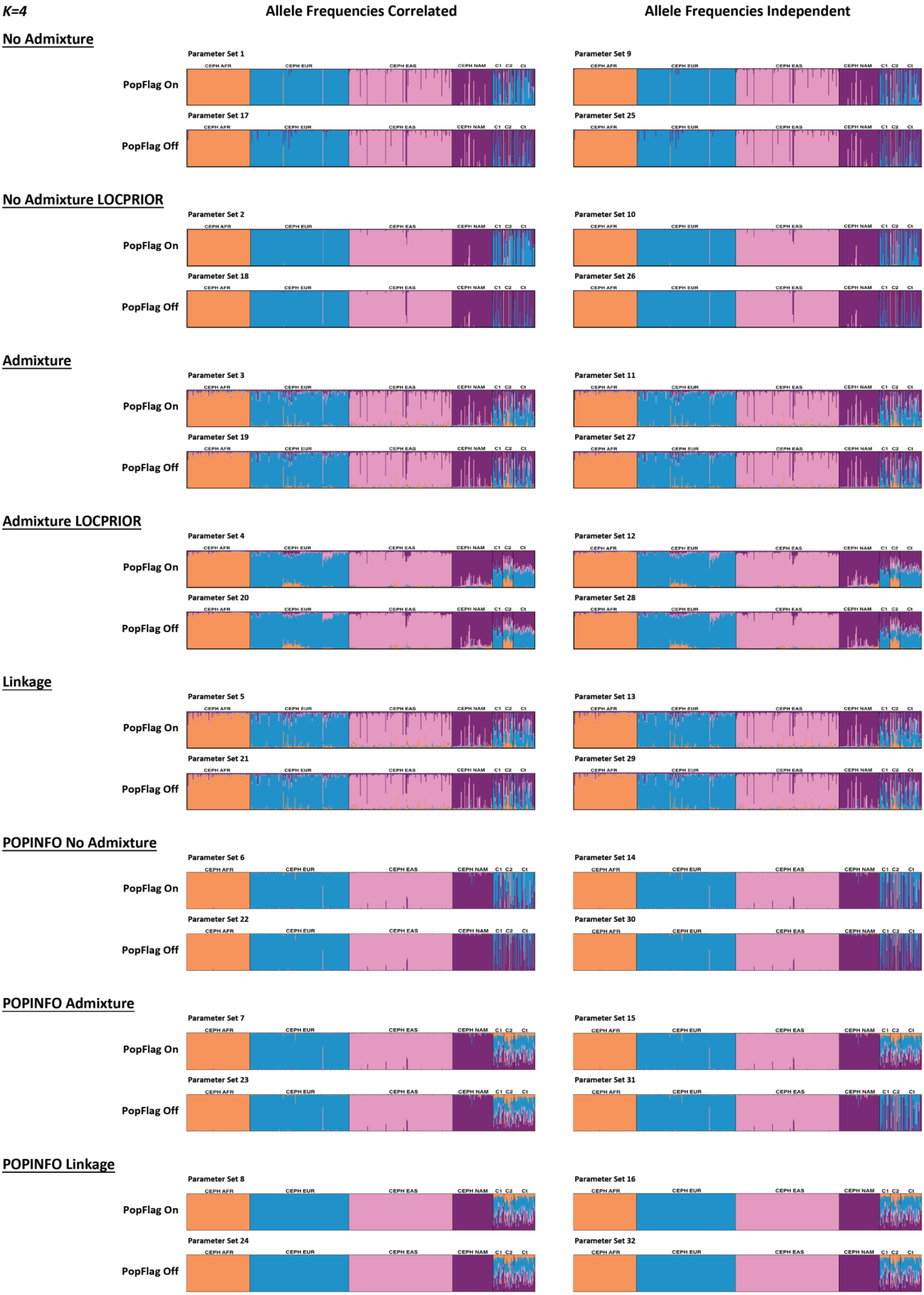
K=4
Allele Frequencies Correlated
Allele Frequencies Independent
No Admixture
Parameter Set 1
Parameter Set 9
PopFlag On
Parameter Set 17
Parameter Set 25
PopFlag Off
No Admixture LOCPRIOR
Parameter Set 2
Parameter Set 10
PopFlag On
Parameter Set 18
Parameter Set 26
PopFlag Off
Admixture
Parameter Set 3
Parameter Set 11
PopFlag On
Parameter Set 19
Parameter Set 27
PopFlag Off
Admixture LOCPRIOR
Parameter Set 4
Parameter Set 12
PopFlag On
Parameter Set 20
Parameter Set 28
PopFlag Off
Linkage
Parameter Set 5
Parameter Set 13
PopFlag On
Parameter Set 21
Parameter Set 29
PopFlag Off
POPINFO No Admixture
Parameter Set 6
Parameter Set 14
PopFlag On
Parameter Set 22
Parameter Set 30
PopFlag Off
POPINFO Admixture
Parameter Set 7
Parameter Set 15
PopFlag On
Parameter Set 23
Parameter Set 31
PopFlag Off
POPINFO Linkage
Parameter Set 8
Parameter Set 16
PopFlag On
Parameter Set 24
Parameter Set 32
PopFlag Off
CEPH AFR
CEPH EUR
CEPH EAS
CEPH NAM
C1
C2
Ct

The optimum *K* value was 4 for almost all parameter sets, although parameter sets 6, 7, 8, 14, 15 and 16 (those corresponding to the *POPINFO* models with *POPFLAG* on) present some signals where the optimum *K* equals 3. Results for *K*=3 are plotted in Fig. 10 as described previously and are presented as a comparison point to the *K*=4 bar plots.

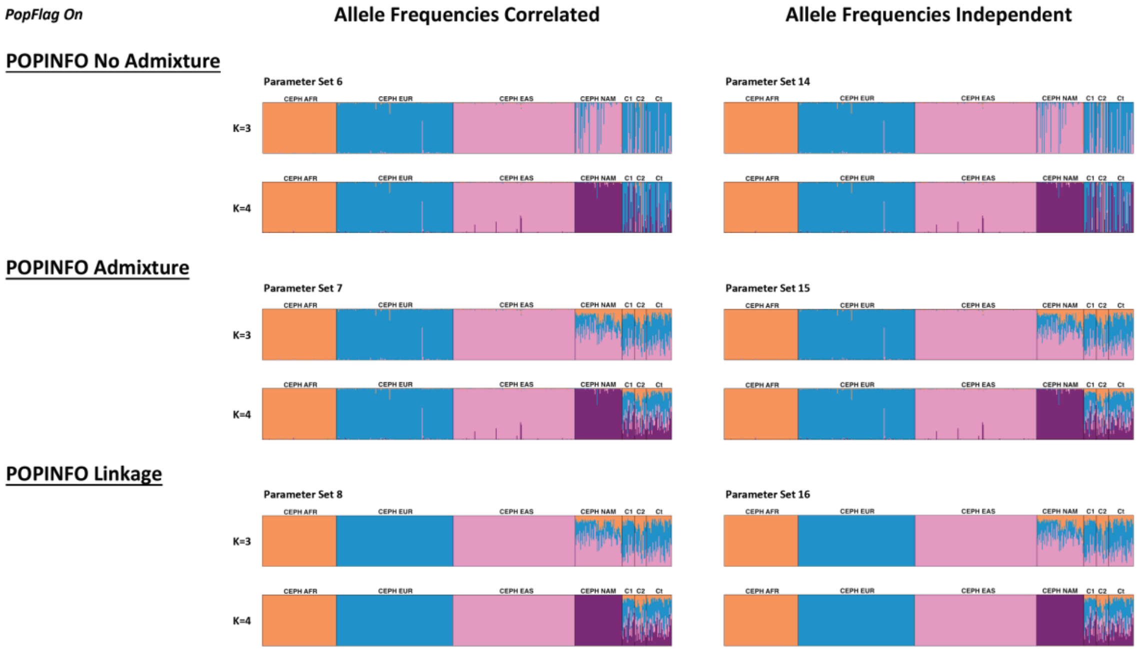

Figure 10: *STRUCTURE* analysis of the test study data set with parameter sets 6, 7, 8, 14, 15 and 16. *K*=3 bar plots are presented for comparison with *K*=4.

The case-control samples were analysed with *STRUCTURE* followed by a *STRAT* test to evaluate if significant differences are discernable between Cases 1 or Cases 2 and the Controls. The *STRUCTURE* analysis was performed with 100,000 *burnin* period and 100,000 MCMC repeats after *burnin* for the *no admixture* and *admixture* models considering the *correlated allele frequencies* model. Each parameter set was analysed with three replicates for *K*=1 to *K*=5. The optimum *K* value was assessed through the analysis of the Ln P(D) distribution plots from *Structure Harvester* (Earl and vonHoldt, 2012). For all analyses the optimum *K* value was equal to 2 – barplots for this *K* value were designed using *CLUMPP* and *distruct*. *STRAT* analysis was performed as described in section 4 using the default parameters. Only results for the *no admixture* model are presented (Fig. 11).

a) **Structure Harvester**

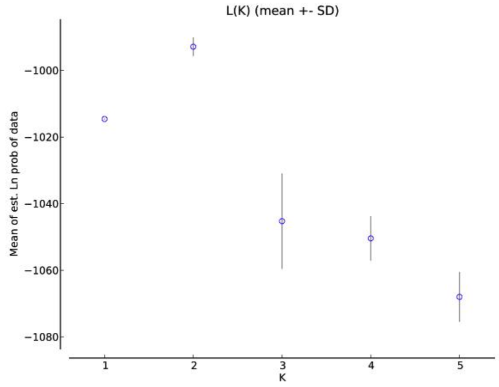

STRUCTURE barplot

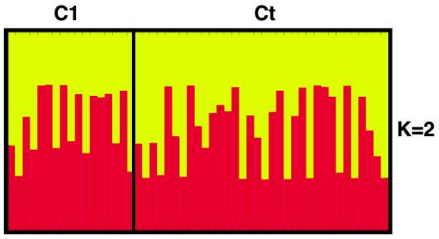

STRAT results

1:	chisq= 0.314	1 df;	TS = 0.45,	p = 6.32000e-001	M1	
2:	allele remained after pooling					
3:	chisq= 1.420	1 df;	TS = 2.12,	p = 1.22000e-001	M3	
4:	chisq= 1.585	1 df;	TS = 2.35,	p = 1.10000e-001	M4	
5:	chisq= 0.000	1 df;	TS = 0.22,	p = 8.11000e-001	M5	
6:	chisq= 0.084	1 df;	TS = 0.10,	p = 9.20000e-001	M6	
7:	chisq= 0.176	1 df;	TS = 0.61,	p = 5.52000e-001	M7	
8:	chisq= 0.000	1 df;	TS = 0.05,	p = 9.51000e-001	M8	
9:	chisq= 0.182	1 df;	TS = 0.90,	p = 3.87000e-001	M9	
10:	chisq= 1.912	1 df;	TS = 0.52,	p = 4.97000e-001	M10	
11:	chisq= 2.863	1 df;	TS = 2.04,	p = 1.24000e-001	M11	
12:	chisq= 6.301	1 df;	TS = 2.85,	p = 4.40000e-002	M12	*
13:	chisq= 0.022	1 df;	TS = 0.04,	p = 9.12000e-001	M13	
14:	chisq= 0.815	1 df;	TS = 0.82,	p = 3.64000e-001	M14	
15:	chisq= 0.495	1 df;	TS = 0.16,	p = 8.52000e-001	M15	
16:	chisq= 0.882	1 df;	TS = 1.03,	p = 2.42000e-001	M16	
17:	chisq= 2.755	1 df;	TS = 2.79,	p = 5.90000e-002	M17	

Summary of distribution of p-values

						Sum	Accum Sum	Obs-Exp
0.00---0.05:	0.000	0.000	0.000	0.000	0.063	0.0625	0.0625	0.0125
0.05---0.10:	0.063	0.000	0.000	0.000	0.000	0.0625	0.1250	0.0250
0.10---0.15:	0.000	0.063	0.125	0.000	0.000	0.1875	0.3125	0.1625
0.15---0.20:	0.000	0.000	0.000	0.000	0.000	0.0000	0.3125	0.1125
0.20---0.25:	0.000	0.000	0.000	0.000	0.063	0.0625	0.3750	0.1250
0.25---0.30:	0.000	0.000	0.000	0.000	0.000	0.0000	0.3750	0.0750
0.30---0.35:	0.000	0.000	0.000	0.000	0.000	0.0000	0.3750	0.0250
0.35---0.40:	0.000	0.063	0.000	0.063	0.000	0.1250	0.5000	0.1000
0.40---0.45:	0.000	0.000	0.000	0.000	0.000	0.0000	0.5000	0.0500
0.45---0.50:	0.000	0.000	0.000	0.000	0.063	0.0625	0.5625	0.0625
0.50---0.55:	0.000	0.000	0.000	0.000	0.000	0.0000	0.5625	0.0125
0.55---0.60:	0.063	0.000	0.000	0.000	0.000	0.0625	0.6250	0.0250
0.60---0.65:	0.000	0.000	0.000	0.063	0.000	0.0625	0.6875	0.0375
0.65---0.70:	0.000	0.000	0.000	0.000	0.000	0.0000	0.6875	-0.0125
0.70---0.75:	0.000	0.000	0.000	0.000	0.000	0.0000	0.6875	-0.0625
0.75---0.80:	0.000	0.000	0.000	0.000	0.000	0.0000	0.6875	-0.1125
0.80---0.85:	0.000	0.063	0.000	0.000	0.000	0.0625	0.7500	-0.1000
0.85---0.90:	0.063	0.000	0.000	0.000	0.000	0.0625	0.8125	-0.0875
0.90---0.95:	0.000	0.063	0.063	0.000	0.000	0.1250	0.9375	-0.0125
0.95---1.00:	0.063	0.000	0.000	0.000	0.000	0.0625	1.0000	0.0000

b) **Structure Harvester**

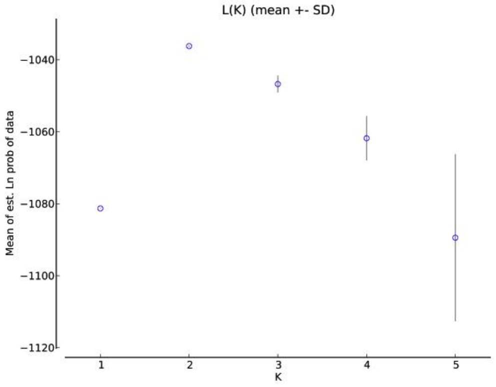

STRUCTURE barplot

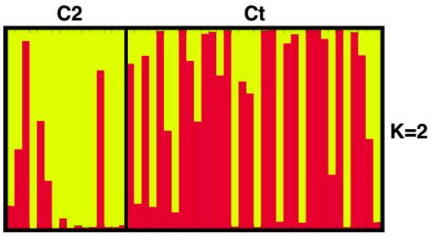

STRAT results

1:	chisq=5.583	1df;	TS=4.71,	p=6.00000e-003	M1	**
2:	chisq=21.140	1df;	TS=9.12,	p=0.00000e+000	M2	***
3:	chisq=0.949	1df;	TS=0.50,	p=6.33000e-001	M3	
4:	chisq=0.563	1df;	TS=2.15,	p=9.40000e-002	M4	
5:	chisq=0.046	1df;	TS=0.53,	p=6.09000e-001	M5	
6:	chisq=1.968	1df;	TS=1.08,	p=3.75000e-001	M6	
7:	chisq=9.631	1df;	TS=5.97,	p=3.00000e-003	M7	**
8:	chisq=0.461	1df;	TS=0.27,	p=7.83000e-001	M8	
9:	chisq=16.185	1df;	TS=4.83,	p=1.10000e-002	M9	*
10:	chisq=0.003	1df;	TS=1.80,	p=1.39000e-001	M10	
11:	chisq=0.085	1df;	TS=0.12,	p=8.86000e-001	M11	
12:	chisq=0.506	1df;	TS=3.39,	p=2.80000e-002	M12	*
13:	chisq=10.725	1df;	TS=0.08,	p=9.22000e-001	M13	
14:	chisq=14.299	1df;	TS=1.90,	p=8.60000e-002	M14	
15:	chisq=0.158	1df;	TS=0.78,	p=5.09000e-001	M15	
16:	chisq=8.316	1df;	TS=0.37,	p=5.07000e-001	M16	
17:	chisq=5.823	1df;	TS=0.16,	p=8.79000e-001	M17	

Summary of distribution of p-values

						Sum	Accum Sum	Obs-Exp
0.00---0.05:	0.176	0.059	0.059	0.000	0.000	0.2941	0.2941	0.2441
0.05---0.10:	0.000	0.000	0.000	0.059	0.059	0.1176	0.4118	0.3118
0.10---0.15:	0.000	0.000	0.000	0.059	0.000	0.0588	0.4706	0.3206
0.15---0.20:	0.000	0.000	0.000	0.000	0.000	0.0000	0.4706	0.2706
0.20---0.25:	0.000	0.000	0.000	0.000	0.000	0.0000	0.4706	0.2206
0.25---0.30:	0.000	0.000	0.000	0.000	0.000	0.0000	0.4706	0.1706
0.30---0.35:	0.000	0.000	0.000	0.000	0.000	0.0000	0.4706	0.1206
0.35---0.40:	0.000	0.000	0.059	0.000	0.000	0.0588	0.5294	0.1294
0.40---0.45:	0.000	0.000	0.000	0.000	0.000	0.0000	0.5294	0.0794
0.45---0.50:	0.000	0.000	0.000	0.000	0.000	0.0000	0.5294	0.0294
0.50---0.55:	0.118	0.000	0.000	0.000	0.000	0.1176	0.6471	0.0971
0.55---0.60:	0.000	0.000	0.000	0.000	0.000	0.0000	0.6471	0.0471
0.60---0.65:	0.059	0.000	0.000	0.059	0.000	0.1176	0.7647	0.1147
0.65---0.70:	0.000	0.000	0.000	0.000	0.000	0.0000	0.7647	0.0647
0.70---0.75:	0.000	0.000	0.000	0.000	0.000	0.0000	0.7647	0.0147
0.75---0.80:	0.000	0.000	0.000	0.059	0.000	0.0588	0.8235	0.0235
0.80---0.85:	0.000	0.000	0.000	0.000	0.000	0.0000	0.8235	-0.0265
0.85---0.90:	0.000	0.000	0.059	0.059	0.000	0.1176	0.9412	0.0412
0.90---0.95:	0.000	0.000	0.059	0.000	0.000	0.0588	1.0000	0.0500
0.95---1.00:	0.000	0.000	0.000	0.000	0.000	0.0000	1.0000	0.0000

Figure 11 A case-control sample analysis presenting Ln P(D) distribution plots, *STRUCTURE* bar plots and *STRAT* table results. a) Case 1 (C1) are compared to the Control (Ct) samples; b) Case 2 (C2) are compared to the Control (Ct) samples.

6. *Discussion and final remarks*

STRUCTURE software allows the comparison of populations taking into account typed genetic variables. For example, it is possible to infer ancestry components of a population or individual with respect to reference populations or, when a phenotypic variable is compared, the *STRAT* algorithm can be applied for the analysis of stratification. The use of a Bayesian method such as *STRUCTURE* offers some advantages especially in the context of the classification of admixed individuals since it allows the use of prior information that can be informative to assist the calculation of ancestry proportions for these individuals. In this way any information on data, the markers selected and the type of analysis desired is relevant before the selection of the analysis parameters before initiating a *STRUCTURE* run.

As described in detail in section 1.4, *STRUCTURE* implements different analysis models whose selection depends on the data and study objectives. Therefore, this guide centres on the effect changes in such models and prior information can have on the results.

The two most contrasting ancestry models are the *no admixture* and *admixture* models. If there is no prior knowledge about the origin of the populations under study or if the user suspects that they are completely discrete then the *no admixture* model is suitable. However admixture between populations is a common characteristic of real data such that individuals may have recent ancestors in more than one population. In these cases knowing the approximate median value of the inherited proportions for each individual and/or population can be important data for the population studies being made. In this case the *admixture* model is more appropriate. These two models can also be used considering the sampling location information (*LOCPRIOR* model) to assist the clustering process made by *STRUCTURE*. This option can be used when there is linguistic, geographical, cultural or other phenotypic information available for the population under study and it is particularly informative when there is a weak structure signal.

Another model, the *linkage* model, is based on the *admixture* model so it can be used under the same assumptions but it was specially designed to deal with admixture linkage disequilibrium, particularly to study admixed populations. This model was presented by Falush et al. (2003) and it allows more accurate estimates of statistical uncertainty when linked loci are used. As for other parameters, it is important to know the markers used – in this particular case information about the degree and context of linkage of the genetic markers typed in the study.

No admixture, *admixture* and *linkage* models can also be analysed as part of the USEPOPINFO model when the data set is very informative. This model uses the sampling locations to test for migrants or hybrids so it should be used with caution – but is appropriate when the user is sure about the sampling locations and they correspond almost exactly with the groups defined by *STRUCTURE*. The disadvantage of the USEPOPINFO model arises with the posterior manipulation of the results. The *individual Q-matrix* comprises probabilities (and not ancestry membership proportions) that are presented in a format that is incompatible with *CLUMPP* or *distruct*.

All the models considered up to now can be used in conjunction with the POPFLAG model. POPFLAG considers specific information about the population of origin of certain reference individuals to help infer the ancestry of individuals with unknown origin. This option should also be used with caution because the selected reference samples are treated as learning samples and the allele frequencies will be based only on these individuals – which can strongly affect the grouping of the unknown individuals. This is an artificial model that assesses the individual probability of being part of a particular population. Such an approach can be useful if the objective is to group individuals/populations in comparison with a well-defined and studied reference data set. When this model is used in conjunction with the USEPOPINFO model the *individual Q-matrix* is composed of two distinct parts: for the individuals with *POPFLAG*=1 the matrix presents probabilities; on the other hand, individuals with *POPFLAG*=0 will be analysed according to the admixture model defined (*no admixture*, *admixture* or *linkage*) and ancestry membership proportions are output.

Concerning the allele frequency model, the *correlated allele frequencies* model is more conservative and should be the preference. The *independent allele frequencies* model requires some knowledge about the correlation levels across populations so there needs to be evidence in the data that allele frequencies show reasonably different distributions in distinct populations. On the other hand, the *correlated allele frequencies* model allows a greater power to detect distinct populations that show very similar allele frequency distributions. However the *correlated allele frequencies* model will give the same results as the *independent allele frequencies* model if there are not high levels of correlation across populations. Therefore using the *correlated allele frequencies* model will guarantee that an unexpected or unknown correlation is detected without affecting the results if no correlation exists.

As a final remark about the models implemented in *STRUCTURE,* all models include specific statistical parameters that give some feedback on the data and information considered, such as *r* (informativeness of the sampling location data), or the more sensitive values, such as *alpha* (relative admixture level between populations) and *lambda* (quantifier of the prior allele frequencies).

Reference populations are important when the user wants to classify study populations, especially admixed populations. On the other hand, if the user is interested in case-control studies there is not a pressing need for ancestral reference populations since *STRUCTURE* compares genetic populations. In fact, when analysing cases and controls, ancestry membership proportions of the two groups are compared through a chi-square test and a *p* value is obtained. If *p* is significant, population stratification is present. However, some authors recommend a replication study in another population, use of other neutral markers to improve the correction and verification of the associations found.

Concerning the example presented (section 5), we used *STRUCTURE* to analyse a unique set of individuals with genotype data and to perform all possible analysis factor

combinations taking into account the optimal *K*. For example, in the *LOCPRIOR* model the *r*-mean values obtained under the different parameters tested were less than one. This indicates that the prior information was useful to assist clustering, taking into account the small number of markers employed for the analysis, particularly for the admixed samples. One advantage of *STRUCTURE* is that it verifies if the prior information is consistent with the genotypic differences or similarities amongst populations before assigning the proportion of ancestry.

The *STRAT* analysis we performed is summarized in Fig. 11. *STRAT* makes a *chi-square* test and calculates a *p*-value for each marker. In this way several tests (the same number as the number of markers) are performed and when any of the resulting *p*-values are smaller than 0.05 the chi-square test is significant. When comparing Cases 1 (C1) with Controls (Ct) only 16 tests were performed since one marker (M2) has a low frequency for allele T (0.070) – *STRAT* pools alleles with fewer than 10 copies. For the comparison of Cases 2 (C2) with the Ct, all 17 markers were analysed. In the summary of the distribution of *p*-values the first line has considerable value for the analysis since it includes the probabilities lower than 0.05 – the expected probability value. Dividing one by the number of analysed markers the user can obtain the probability value for each significant marker. In our example comparing C1 to Ct, as only 16 markers were considered, the probability of finding a significant p-value is 0.063 (while comparing C2 to Ct with 17 markers, this probability is 0.059). Depending on the number of markers that fall into each p-value range, the expected probability is multiplied by the number of markers found. For example in the study data of Fig. 11 used to illustrate *STRAT*, in the C1-Ct comparison there is only one significant marker (M12) with a *p*-value of $4.4x10^{-2}$ – that is why the third column (0.02-0.03 range) has a value of 0.063. In the C2-Ct comparisons there are five significant markers, three of them with *p*-values falling into the 0.00-0.01 range – so the first column has a value of 0.176 (3x0.059). The total sum value of each probability fraction (the observed probability value) is compared with the expected probability. For the first fraction this value is 0.05 but the value increases with the other fractions (0.10 for the second fraction, 0.15 for the third, etc.). The resulting value (the last column of the table) indicates if multiple correction is a concern: positive values here illustrate the fact that observed probabilities are more frequent than expected. Briefly, *STRAT* offers corrected *p*-values at any given locus taking into account the confounding effects of population stratification.

With this article we present an updated review and some recommendations that can be helpful when performing population structure and admixture association analyses. We focused our study on *STRUCTURE* and its associated post-hoc analysis software (*CLUMPP*, *distruct* and *STRAT*). A simulated example file was thoroughly analyzed with a wide range of parameter combinations. The concluding remark it is important to make after these studies is that there is not one standard analysis parameter in *STRUCTURE* – the data and the study objectives will influence the choice of the most appropriate parameter – therefore precaution should be applied in order to avoid overestimating the population structure present in complex data.

7. References

Bouakaze, C., Keyser, C., Crubezy, E., Montagnon, D., and Ludes, B. (2009). Pigment phenotype and biogeographical ancestry from ancient skeletal remains: inferences from multiplexed autosomal SNP analysis. *Int J Legal Med* 123**,** 315-325.

Corander, J., Waldmann, P., and Sillanpaa, M.J. (2003). Bayesian Analysis of Genetic Differentiation Between Populations. *Genetics* 163**,** 367-374.

Earl, D.A., and Vonholdt, B.M. (2012). STRUCTURE HARVESTER: a website and program for visualizing STRUCTURE output and implementing the Evanno method. *Conservation Genetics Resources* 4 (2)**,** 359-361.

Evanno, G., Regnaut, S., and Goudet, J. (2005). Detecting the number of clusters of individuals using the software STRUCTURE: a simulation study. *Mol Ecol* 14**,** 2611-2620.

Falush, D., Stephens, M., and Pritchard, J.K. (2003). Inference of Population Structure Using Multilocus Genotype Data: Linked Loci and Correlated Allele Frequencies. *Genetics* 164**,** 1567-1587.

Hubisz, M.J., Falush, D., Stephens, M., and Pritchard, J.K. (2009). Inferring weak population structure with the assistance of sample group information. *Molecular Ecology Resources* 9**,** 1322-1332.

Jakobsson, M., and Rosenberg, N.A. (2007). CLUMPP: a cluster matching and permutation program for dealing with label switching and multimodality in analysis of population structure. *Bioinformatics* 23**,** 1801-1806.

Patterson, N., Price, A.L., and Reich, D. (2006). Population structure and eigenanalysis. *PLoS Genet* 2**,** e190.

Pfaff, C.L., Parra, E.J., Bonilla, C., Hiester, K., Mckeigue, P.M., Kamboh, M.I., Hutchinson, R.G., Ferrell, R.E., Boerwinkle, E., and Shriver, M.D. (2001). Population structure in admixed populations: effect of admixture dynamics on the pattern of linkage disequilibrium. *Am J Hum Genet* 68**,** 198-207.

Pritchard, J.K., and Rosenberg, N.A. (1999). Use of Unlinked Genetic Markers to Detect Population Stratification in Association Studies. *The American Journal of Human Genetics* 65**,** 220-228.

Pritchard, J.K., Stephens, M., and Donnelly, P. (2000a). Inference of population structure using multilocus genotype data. *Genetics* 155**,** 945-959.

Pritchard, J.K., Stephens, M., Rosenberg, N.A., and Donnelly, P. (2000b). Association mapping in structured populations. *Am J Hum Genet* 67**,** 170-181.

Rosenberg, N.A., Mahajan, S., Ramachandran, S., Zhao, C., Pritchard, J.K., and Feldman, M.W. (2005). Clines, clusters, and the effect of study design on the inference of human population structure. *PLoS Genet* 1**,** e70.
